# Ribosomal Readthrough at a Short UGA Stop Codon Context Triggers Dual Localization of Metabolic Enzymes in Fungi and Animals

**DOI:** 10.1371/journal.pgen.1004685

**Published:** 2014-10-23

**Authors:** Alina C. Stiebler, Johannes Freitag, Kay O. Schink, Thorsten Stehlik, Britta A. M. Tillmann, Julia Ast, Michael Bölker

**Affiliations:** 1 Department of Biology, Philipps-University Marburg, Marburg, Germany; 2 LOEWE Excellence Cluster for Integrative Fungal Research (IPF), Senckenberg Society, Frankfurt am Main, Germany; 3 Faculty of Medicine, Centre for Cancer Biomedicine, University of Oslo, Montebello, Oslo, Norway; 4 Department of Biochemistry, Institute for Cancer Research, Oslo University Hospital, Montebello, Oslo, Norway; 5 LOEWE Center for Synthetic Microbiology (SYNMIKRO), Marburg, Germany; University of Kent, United Kingdom

## Abstract

Translation of mRNA into a polypeptide chain is a highly accurate process. Many prokaryotic and eukaryotic viruses, however, use leaky termination of translation to optimize their coding capacity. Although growing evidence indicates the occurrence of ribosomal readthrough also in higher organisms, a biological function for the resulting extended proteins has been elucidated only in very few cases. Here, we report that in human cells programmed stop codon readthrough is used to generate peroxisomal isoforms of cytosolic enzymes. We could show for NAD-dependent lactate dehydrogenase B (LDHB) and NAD-dependent malate dehydrogenase 1 (MDH1) that translational readthrough results in C-terminally extended protein variants containing a peroxisomal targeting signal 1 (PTS1). Efficient readthrough occurs at a short sequence motif consisting of a UGA termination codon followed by the dinucleotide CU. Leaky termination at this stop codon context was observed in fungi and mammals. Comparative genome analysis allowed us to identify further readthrough-derived peroxisomal isoforms of metabolic enzymes in diverse model organisms. Overall, our study highlights that a defined stop codon context can trigger efficient ribosomal readthrough to generate dually targeted protein isoforms. We speculate that beyond peroxisomal targeting stop codon readthrough may have also other important biological functions, which remain to be elucidated.

## Author Summary

Eukaryotic organisms use various strategies to generate protein isoforms with different function or intracellular localization from a single gene. While differential splicing of mRNA is the most common mechanism to expand the number of encoded proteins, translational readthrough of stop codons is an alternative strategy to create protein variants with C-terminally extended proteins. Recently, it has been shown that fungi use both alternative splicing and translational readthrough to specify peroxisomal isoforms of glycolytic enzymes. Here we show that stop codon readthrough is also used in the animal kingdom to target important metabolic enzymes to peroxisomes. Interestingly, several of these enzymes have a function in peroxisomal redox homeostasis and energy metabolism. It has been described that termination fidelity is modulated by oxidation of specific ribosomal proteins. This suggests that dual targeting via translational readthrough allows adaptation of peroxisomal metabolism to the oxidative status of the cell.

## Introduction

Although translation of mRNA usually occurs with high fidelity, different recoding mechanisms exist that alter the amino acid sequence of the resulting polypeptide [1], [2]. Programmed ribosomal frameshifting and stop codon readthrough are commonly used to maximize genomic coding capacity in viruses [3]. Bacteria containing suppressor tRNAs and *Saccharomyces cerevisiae* strains expressing a non-functional prion form of the eukaryotic release factor 3 display significantly enhanced bypass of stop codons [4], [5]. This results in C-terminal extension of many proteins and increases phenotypic variability. Stop codon readthrough of individual genes was first described for viral replicases in bacteriophage Qβ and tobacco mosaic virus [6]–[9]. In their pro- and eukaryotic hosts, however, only a few cellular genes have been identified, where a biological function could be attributed to readthrough derived extended polypeptides [2], [10], [11], [12]. Although in mammals readthrough derived isoforms have been detected for beta-hemoglobin [13] and for myelin P0 [14], it is unknown whether the derived C-terminal extensions are functionally important. Recently, bioinformatic analysis and ribosome profiling revealed evidence for abundant and regulated bypass of termination in different developmental stages of *Drosophila* and other metazoa, suggesting a conserved function for translational readthrough in animals [15], [16].

We have recently reported that translational readthrough and alternative splicing are used in fungi to generate peroxisomal isoforms of several glycolytic enzymes [17]. Peroxisomes are single-membrane compartments with a major role in fatty acid degradation [18], [19]. In humans, peroxisomes are essential and peroxisome disorders cause severe syndromes [20]–[22]. The majority of proteins destined for the peroxisomal matrix contain a type 1 peroxisomal targeting signal (PTS1), a short C-terminal motif derived from the prototypical sequence Ser-Lys-Leu (SKL) [23], [24]. Here, we report that translational readthrough at a short conserved stop codon context is used in animals and fungi to generate peroxisomal isoforms of important metabolic enzymes.

## Results/Discussion

### A UGA stop codon followed by the dinucleotide CU serves as a core element for efficient translational readthrough in fungi

**Figure 1:**
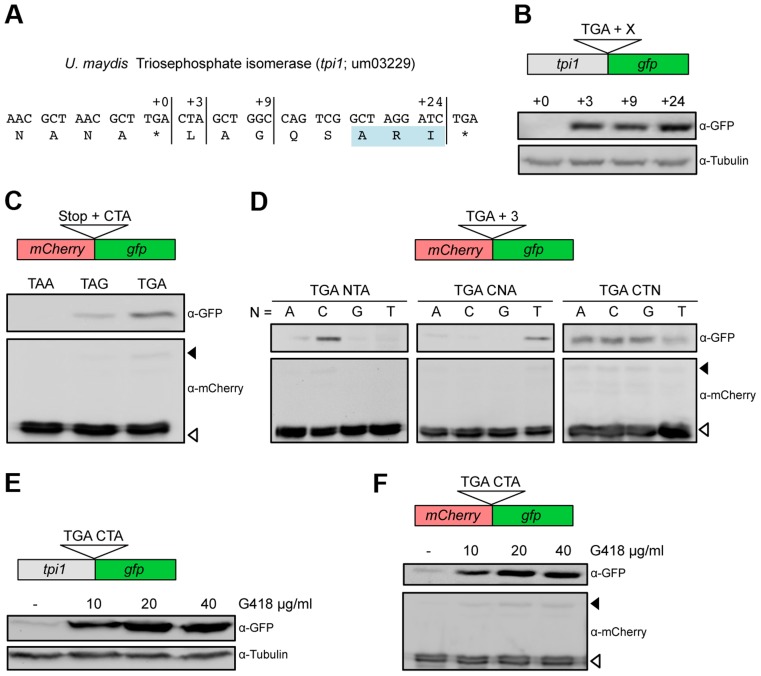
Characterization of sequence requirements for translational readthrough in *U. maydis*. (A) 3′ sequence of the *U. maydis tpi1* gene and its translation. PTS1 is highlighted. (B) Tpi1 with indicated C-terminal extensions was fused to GFP and analyzed by Western blot. (C) A reporter construct consisting of *mCherry* and *gfp* interrupted by a stop codon was expressed. Stop codon readthrough was analyzed by detection of GFP by Western blot. The termination codon TGA was replaced by TAA or TAG and tested in combination with the downstream sequence CTA for ribosomal readthrough. (D) Nucleotides downstream of TGA were exchanged as indicated and tested as described in (C) for stop codon readthrough. (E) Tpi1 including TGA CTA was fused to GFP. Cells were incubated with different amounts of G418 and analyzed for readthrough by Western blotting. (F) TGA CTA was inserted between *mCherry* and *gfp* of the reporter construct. Cells were incubated with different amounts of G418 and analyzed for readthrough by Western blotting. Filled arrows indicate readthrough products while blank arrows indicate products terminated at the first stop codon.

We have previously shown that in the fungus *Ustilago maydis*, a PTS1 containing isoform of triosephosphate isomerase (Tpi1) is generated by stop codon readthrough (Fig. 1A) [17]. To determine the minimal sequence requirements for efficient readthrough, we fused the reporter gene g*fp* (green fluorescent protein) at different positions downstream of the *tpi1* stop codon (Fig. 1B). Western analysis revealed that three nucleotides (CUA) following the UGA stop codon were sufficient to trigger efficient stop codon readthrough (Fig. 1B). Readthrough was also observed if this sequence motif was inserted between two reporter genes (Fig. 1C). Stop codon identity was found to be important as replacement of UGA by UAG or UAA reduced translational readthrough (Fig. 1C). Mutational analysis revealed that the identity of the first and the second nucleotide following the stop codon mainly determine readthrough efficiency (Fig. 1D). Thus, a UGA stop codon followed by the dinucleotide CU is sufficient to trigger efficient translational readthrough in *U. maydis*. Readthrough cannot result from incorporation of the non-canonical amino acid selenocysteine at the UGA termination codon since fungi do not contain the required specialized translation machinery [2]. It remains to be elucidated which near-cognate tRNA is required for recoding of the UGA stop codon within the UGA CU context. It has already been shown that tryptophan, arginine, cysteine or serine can be incorporated at UGA stop codons [25], [26]. Aminoglycoside antibiotics inhibit protein biosynthesis by reducing the accuracy of ribosomal translation. They also cause misreading of termination codons and thus enhance the level of ribosomal readthrough [27], [28], [29]. If the aminoglycoside G418 was added to growing *U. maydis* cells we observed a concentration dependent increase of C-terminally extended fusion proteins, for both Tpi1 and the readthrough reporter construct (Fig. 1E and F).

**Figure 2:**
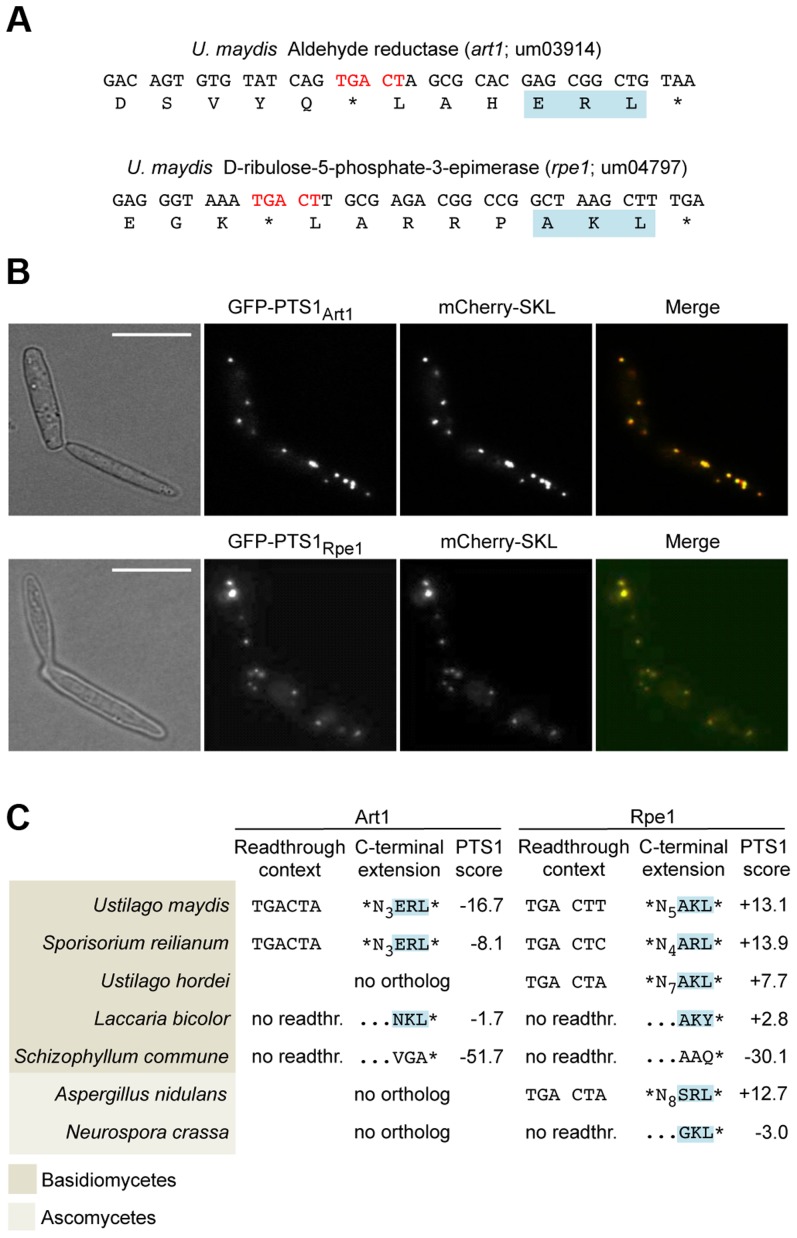
Peroxisomal targeting of NADH-dependent aldehyde reductase Art1 and D-ribulose-5-phosphate-3-epimerase Rpe1 is conserved in fungi. (A) 3′ sequences of *U. maydis* genes *art1* and *rpe1* together with their derived polypeptide sequence. PTS1 and readthrough elements are highlighted. All accession numbers are from MIPS *Ustilago maydis* DataBase (MUMDB). (B) The PTS1 motifs of Art1 and Rpe1 were fused to GFP and co-expressed with the peroxisomal marker mCherry-SKL in *U. maydis* cells. BF indicates bright field. Scale bars represent 10 µm. (C) Conservation of readthrough derived peroxisomal isoforms of Art1 and Rpe1 orthologs in fungi. Predictions were carried out with a PTS1 predictor [66]. In some organisms the PTS1 is part of the original open reading frame indicated by no readthr. and …NNN*.

To identify additional *U. maydis* genes coding for proteins with readthrough derived PTS1 motifs we screened all open reading frames ending on TGA that are followed by CT (Tab. S1). In addition to the previously described glycolytic enzymes with cryptic PTS1 motifs [17], we identified D-ribulose-5-phosphate-3-epimerase and an NADH-dependent aldehyde reductase (Fig. 2A). We could demonstrate that the readthrough derived extensions of both proteins trigger peroxisomal targeting in *U. maydis* (Fig. 2B). While the biological function of the aldehyde reductase is yet unknown, D-ribulose-5-phosphate-3-epimerase is an enzyme of the pentose phosphate pathway. Other components of this pathway have already been described to be located in peroxisomes [30]–[32]. Genome comparison revealed that peroxisomal targeting of both enzymes via ribosomal readthrough at UGA CU occurs also in other fungal species (Fig. 2C).

### Translational readthrough at UGA CU in human cells

**Figure 3:**
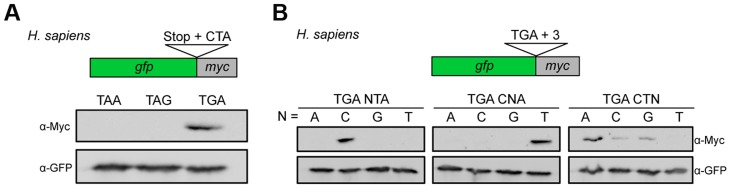
Characterization of sequence requirements for translational readthrough in human cells. (A) A reporter construct consisting of *gfp* and *myc* interrupted by a stop codon was expressed in HeLa cells. Stop codon readthrough was analyzed by detection of the myc-epitope by Western blot. The termination codon TGA was replaced by TAA or TAG and tested in combination with the downstream sequence CTA for ribosomal readthrough. (B) Nucleotides downstream of TGA were exchanged as indicated and tested as described in (A) for stop codon readthrough.

It has been described previously that nucleotides downstream of the termination codon influence termination efficiency in pro- and eukaryotes [2], [33], [34]. In the mammalian Sindbis virus the motif UGA C is important to trigger stop codon readthrough in vitro [35]. Remarkably, a similar but extended stop codon context was found to suppress the lethal phenotype of a nonsense mutation in a human patient [36]. Indeed, we were able to show that the UGA CU motif identified in *U. maydis* also triggered efficient readthrough in HeLa cells (Fig. 3A). We observed a similar dependence on the identity of the stop codon and the two downstream nucleotides (Fig. 3A and B). An adenine residue at position +3 further stimulated readthrough efficiency (Fig. 3B). Taken together this suggests that the short sequence motif UGA CU(A) promotes stop codon readthrough in a wider range of species.

### Readthrough derived peroxisomal isoforms of human lactate dehydrogenase B (LDHB) and cytosolic malate dehydrogenase (MDH1)

**Figure 4:**
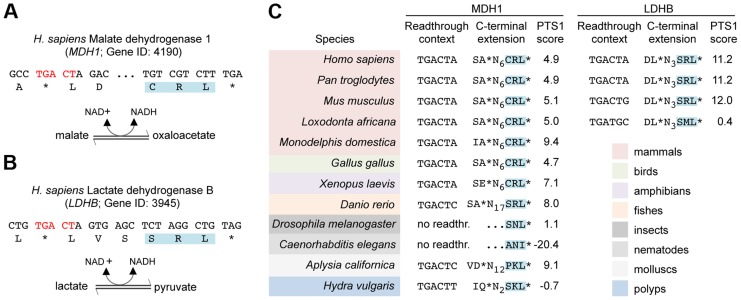
Translational readthrough derived PTS1 motifs of MDH1 and LDHB are conserved in animals. (A) 3′ sequences of the human *MDH1* and its translation. The readthrough element and the predicted PTS1 motifs are highlighted. Enzymatic reactions catalyzed by MDH1 is indicated. NCBI Gene IDs is shown. (B) Human *LDHB* and its translation as in (A). (C) Phylogenetic conservation of readthrough derived MDH1 and LDHB peroxisomal isoforms is indicated by colouring. PTS1 predictions were carried out with PTS1 predictor [66]. PTS1 motifs of MDH1 homologs encoded by the original open reading frame are indicated by no readthr. and …NNN*.

Next, we screened all human protein coding genes ending on TGA CT for potential peroxisomal isoforms generated by stop codon readthrough. To exclude false positives arising from chance occurrence of putative PTS1 motifs, all candidate genes were analyzed for phylogenetic conservation of both stop codon context and readthrough dependent PTS1 (Tab. S2). This analysis revealed two striking candidates, which were characterized in greater detail: cytosolic NAD-dependent malate dehydrogenase 1 (MDH1) (Fig. 4A) and NAD-dependent lactate dehydrogenase B (LDHB) (Fig. 4B). Both enzymes are critical for intracellular redox homeostasis and participate in redox-shuttling across organellar membranes [17], [37]–[39]. Accordingly, these enzymes are generally found in different cellular compartments. Lactate dehydrogenase activity has been detected in rat liver peroxisomes and was found to be required for regeneration of NAD^+^ [40]. MDH1 was recently identified as a peroxisomal protein in human liver cells [41]. However, the import mechanism of both enzymes is not obvious. Our data suggest that peroxisomal isoforms of MDH1 and LDHB containing a PTS1 motif are generated by stop codon readthrough. We confirmed targeting of the predicted peroxisomal isoforms of MDH1 and LDHB by fluorescence microscopy of GFP fusion proteins in HeLa cells (Fig. S1). Phylogenetic analysis revealed that readthrough dependent peroxisomal localization of LDHB is conserved in eutherian mammals (Fig. 4C). Remarkably, the cryptic PTS1 motif of MDH1 is highly conserved in the animal kingdom and occurs already in the cnidarian *Hydra vulgaris* (Fig. 4C).

### Dual targeting of LDHB and MDH1 to peroxisomes and the cytosol in human cells

**Figure 5:**
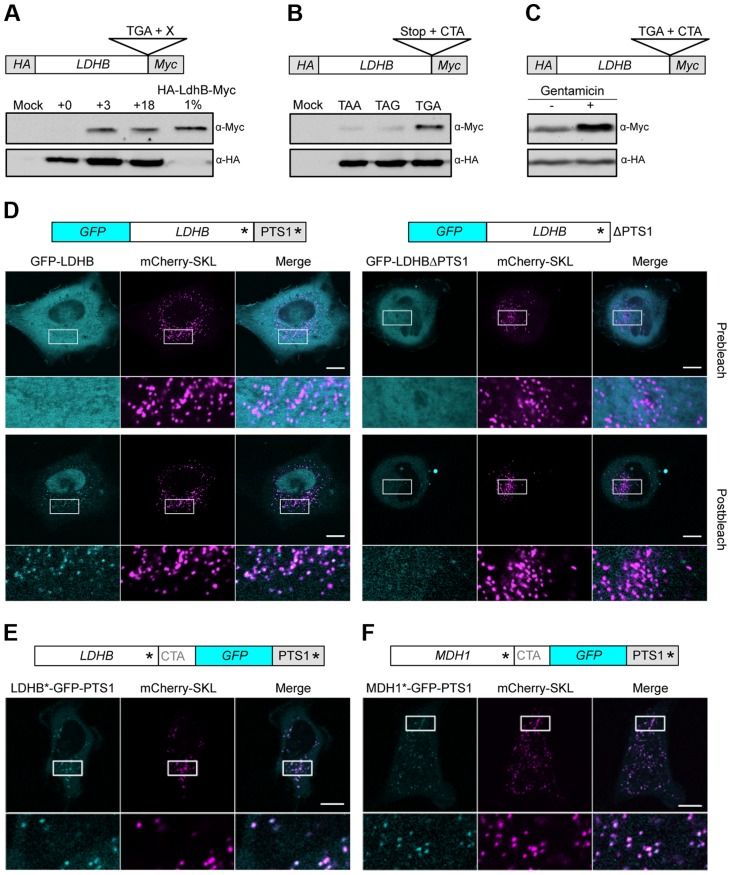
Analysis of peroxisomal targeting of LDHB via translational readthrough in HeLa cells. (A) Western blot analysis of LDHB derivatives fused to HA and Myc. (B) The stop codon of the LDHB construct described in (A) was exchanged by TAA and TAG. (C) Western Blot analysis of LDHB fused to HA and Myc in dependence on Gentamicin (+). (D) GFP-LDHB and GFP-LDHBΔPTS1 were co-expressed with the peroxisomal marker mCherry-SKL. (E) LDHB*-GFP-PTS1 was co-expressed with mCherry-SKL. (F) MDH1*-GFP-PTS1 was co-expressed with mCherry-SKL. Asterisks mark the positions of stop codons. PTS1 indicates the peroxisomal targeting signal downstream of the conventional stop codon of LDHB and MDH1, respectively. Intracellular localization was followed by fluorescence microscopy before and after repeated photobleaching. Magnified areas are indicated and shown below the micrographs. Scale bars represent 10 µm.

Ribosome profiling experiments in human foreskin fibroblasts revealed evidence for stop codon readthrough for *MDH1* but not for *LDHB* [16]. We used HeLa cells to address ribosomal readthrough of full-length LDHB harboring both an N-terminal HA-tag and a C-terminal Myc-tag (Fig. 5A and B). The latter epitope is only generated upon ribosomal readthrough. Leaky termination again depended on the identity of both the UGA stop codon and the nucleotides immediately downstream (Fig. 5A and B). Quantification of readthrough revealed only a small fraction (approx. 1%) of C-terminally extended proteins (Fig. 5A). However, this amount is sufficient to yield a similar protein concentration in peroxisomes and the cytosol as peroxisomes comprise only a small fraction (1–2%) of the cellular volume [42]. To further confirm that the C-terminal extensions are generated by stop codon readthrough we analyzed the quantity of the extended LDHB isoform in the presence of the aminoglycoside gentamicin. We detected an increased amount of the extended LDHB isoform corroborating that it is generated by ribosomal readthrough (Fig. 5C).

To determine the intracellular localization of MDH1 and LDHB we monitored full-length N-terminal GFP-fusion proteins in HeLa cells by live cell imaging. Strong cytosolic fluorescence prevented direct observation of peroxisomal LDHB and MDH1 isoforms (Fig. 5D and S2). However, depletion of cytosolic GFP-LDHB and GFP-MDH1 by repeated photobleaching [43] unveiled peroxisomal targeting of both enzymes (Fig. 5D and S2). Deletion of the PTS1 containing extension abolished peroxisomal localization demonstrating that intracellular sorting of both enzymes depends on ribosomal readthrough (Fig. 5D and S2).

To confirm these data we inserted *GFP* between the TGA CTA motif and the PTS1 encoding sequence of either *LDHB* or *MDH1*. Microscopic analysis confirmed the presence of readthrough derived GFP fusion proteins that predominantly co-localize with the peroxisomal marker mCherry-SKL (Fig. 5E and F).

### Peroxisomal targeting of additional central metabolic enzymes via translational readthrough in animals

**Figure 6:**
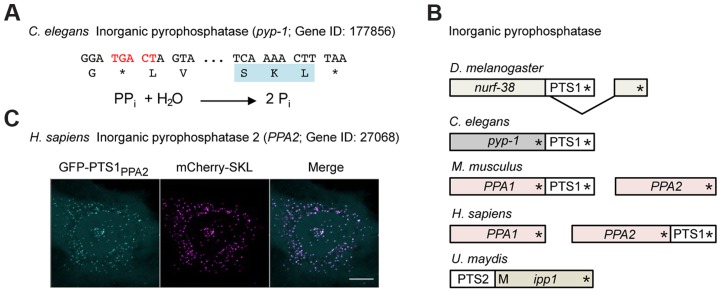
Inorganic pyrophosphatase is targeted to peroxisomes in different species by various mechanisms. (A) 3′ sequence of the *C. elegans pyp-1* gene encoding inorganic pyrophosphatase (Gene ID: 177856) and its translation. The readthrough element and the PTS1 are highlighted. The reaction catalyzed by inorganic pyrophosphatase is shown. (B) Schematic drawings illustrating different mechanisms leading to peroxisomal isoforms of inorganic pyrophosphatase in a variety of species. Asterisks mark the positions of stop codons. M represents an alternative methionine start codon. (C) The readthrough derived PTS1 motif of human PPA2 was fused to GFP and analyzed for peroxisomal localization in HeLa cells. Scale bar represents 10 µm.

We identified additional interesting candidate genes also in other model organisms. *D. melanogaster* NADP-dependent isocitrate dehydrogenase and *Caenorhabditis elegans* inorganic pyrophosphatase contain both the stop codon context UGA CU and a predicted PTS1 in the readthrough derived extension. (Fig. 6A, S3A and S3B; Tab. S2). Interestingly, mammalian and fungal orthologs of NADP-dependent isocitrate dehydrogenase contain a PTS1 motif at their regular C-termini (Fig. S3B and S3C) [44]. Peroxisomal localization of NADP-dependent isocitrate dehydrogenase has already been demonstrated in the fungi *Aspergillus nidulans* and *S. cerevisiae* [45], [46], [47].

The predicted peroxisomal targeting of inorganic pyrophosphatase was found to depend on a variety of molecular mechanisms in different species (Fig. 6B). While alternative splicing generates a C-terminal PTS1 in *D. melanogaster* Nurf-38, the *U. maydis* Ipp1 enzyme contains an N-terminal PTS2 signal (Fig. 6B). Mammals contain two genes coding for cytosolic and mitochondrial versions of inorganic pyrophosphatase (*PPA1* and *PPA2*). Putative readthrough derived PTS1 motifs could be identified in either of these genes depending on the organism (Fig. 6B and C). Inorganic pyrophosphatase is an essential enzyme that drives the direction of all biochemical reactions in which pyrophosphate is generated, e.g. DNA and RNA polymerization [48]. Activity of this enzyme has already been detected in isolated rat liver peroxisomes, where it might be important for ATP dependent activation of fatty acids [49].

### Conclusions

In this study, we have shown that the short readthrough core motif UGA CU(A) is widely used for generation of C-terminally extended proteins. Recently, readthrough derived isoforms of four other mammalian proteins were reported [50]. All corresponding genes contain a TGA CTA motif but readthrough efficiency was found to be further increased by downstream sequences. The presence of a guanine nucleotide at position +4 was of particular importance. We did not test the effect of this residue systematically but noticed that many of the genes identified in our study contain a G at this position (see Fig. 1, 2 and 4). This suggests that the extended motif UGA CUA (G) represents a weak context for termination of translation. While in some cases the amount of readthough product generated by this context alone might be sufficient, higher levels of readthrough require downstream mRNA sequences, which often form secondary structures [51]. Such structural elements might also be involved in efficient readthrough of cellular and viral genes that lack the TGA CT context [1], [3], [16], [17]. A genome wide study in *D. melanogaster* has shown that in genes subject to readthrough secondary structures occur more often if the corresponding stop codons are TAA or TAG [15]. Both stop codons are known to be less leaky than TGA [52].

Another property of the UGA CU(A) element might be relevant for its function in generating peroxisomal isoforms. Recently, it was shown that hydroxylation of a proline residue in the decoding center of ribosomes regulates translational fidelity in response to oxygen availability [53], [54]. Mutational analysis revealed that hydroxylation decreased termination efficiency if the stop codon was followed by a cytosine residue [37]. Thus, intracellular sorting of enzymes targeted to peroxisomes via translational readthrough at the conserved UGA CU motif is likely to be regulated by oxygen levels, which are critical for peroxisomal β-oxidation [30], [55]. Therefore, we speculate that the use of specific readthrough signals allows differential regulation to modulate protein function in response to physiological conditions.

## Materials and Methods

### Strains, growth conditions and transformation

*Escherichia coli* strain TOP10 (Invitrogen) was used for all cloning purposes and amplification of plasmid DNA. All *U. maydis* strains used and created in this study are derivatives of strain Bub8 [56] and listed in table S3. Liquid cultures of *U. maydis* were grown either in YEPS_light_ medium [57] or yeast nitrogen base medium (YNB; Difco) supplemented with 2% glucose at 28°C. *E. coli* and *U. maydis* cells were transformed as described previously [56], [58]. To analyze the effect of G418 on readthrough levels cells of the *U. maydis* strains Bub8 mCherry-TGACTA-GFP and Bub8 TPI+3-GFP were grown overnight and diluted to reach OD_600_ 0,5 after 2 h of growth. At this point 0 µg/ml, 10 µg/ml, 20 µg/ml or 40 µg/ml G418 (Roth) were added to the cultures. After 6 hours the cells were harvested and protein isolation and Western blotting was performed as described below.

### Human cell culture

HeLa cells were kindly provided by Lucie Sauerhering (Institute for Virology, Marburg). The cells were maintained in Dulbecco's modified Eagle's medium (DMEM, Life Technologies) supplemented with 10% fetal bovine serum (FBS, Life Technologies) and 1% antibiotics (10.000 units/ml Penicillin, 10.000 µg/ml Streptomycin, Life Technologies) at 37°C, 5% CO_2_. For transfection, the cells were seeded on 6-well plates and transfected the next day using Lipofectamine2000 (Life Technologies) according to the manufacturer's protocol. 24 h after transfection protein extracts were prepared with RIPA-buffer (50 mM Tris-HCl, pH 7,5; 1% NP-40; 0.25% sodium deoxycholate; 150 mM NaCl; 1 mM EDTA) supplemented with protease inhibitors (Complete, Roche) and phosphatase inhibitors (Phosphatase inhibitor cocktail 2, Sigma-Aldrich). Post nuclear supernatants were stored at −80°C and used for Western blot analysis.

To analyze the effect of aminoglycosides on readthrough levels HeLa cells were transfected as described above. 24 h after transfection the medium was replaced with DMEM supplemented with 10% FBS, 1% Pen/Strep and 800 µg/ml Gentamicin. 48 h after transfection protein isolation and Western blotting was performed as described.

### Molecular cloning and DNA procedures

Standard procedures were followed for all DNA manipulations [59]. Plasmids used for expression analysis in *U. maydis* are derivatives of the plasmid otef-Ala_6_-MMXN [60]. Oligonucleotides and resulting plasmids are listed in table S4. Genomic DNA from *U. maydis* was prepared as described previously [61]. Plasmids used for human cell culture are derivatives of plasmids pEGFP-C1 (Clontech), pEGFP-N1 (Clontech), pmCherry-C1 (Clontech) and pcDNA3.1+ (Invitrogen). All DNA-fragments were inserted between the EcoRI and BamHI sites. cDNA clones encoding human LDHB and MDH1 are from Origene. All plasmids were verified by sequencing.

### Protein isolation and western blotting

For protein isolation, *U. maydis* strains were grown to mid-log phase in YEPS_light_. 10 ml of cells were centrifuged for 5 min at 4000 rpm. The pellet was resuspended in 200 µl TBS containing 0.1% (v/v) Triton-×100 and 1% protease inhibitor cocktail for use with fungal extracts (Sigma) and supplemented with glass beads. Suspensions were frozen to −80°C and disrupted by 30 min incubation on a vibrax shaker (IKA) at 4°C. Suspensions were centrifuged for 15 min at 12000 rpm at 4°C. Supernatants were quantified by a Bradford assay (Biorad). Preparation of proteins from human cell culture was performed as described (see Human cell culture). For protein detection Western blotting analysis was performed. Proteins were separated by SDS-PAGE and blotted on polyvinylidene difluoride membranes or nitrocellulose membranes. Antibodies used in this study: anti-GFP mouse monoclonal (Santa Cruz), anti-mCherry mouse monoclonal (Abcam), anti-α-Tubulin mouse monoclonal (Calbiochem), anti-HA mouse monoclonal (Sigma Aldrich) and anti-Myc mouse monoclonal (New England Biolabs) and secondary antibody (HRP-conjugated goat anti-mouse, Santa Cruz). Detection was performed with Supersignal West Femto and Supersignal West Pico Chemiluminescent Substrate (Thermo Fisher Scientific) and a ChemoCam Imager (INTAS Science Imaging). Western blots were quantified using ImageJ [62].

### Microscopy

*U. maydis* cells from logarithmically growing cultures (YNB-medium) were placed on agarose cushions and visualized by phase contrast (PC) and epifluorescence microscopy using a Zeiss Axiovert 200 microscope. Images were taken using a cooled CCD camera (Hamamatsu Orca-ER) with an exposure time of 30–300 ms. Image acquisition was performed using Improvision Volocity software and processing was carried out with ImageJ.

For microscopic analysis, HeLa cells were grown on Glass Bottom dishes (Mattek) in DMEM medium supplemented with 10% FCS. Cells were transfected with equal amounts (1 µg of total DNA) of the tested construct and the peroxisomal marker mCherry-SKL using Fugene6 reagent (Promega). Prior to imaging, cells were shifted to Life Cell Imaging buffer (Life Technologies) and kept at 37°C. Life cell imaging and photobleaching experiments were performed using a LSM710 confocal microscope (Zeiss) equipped with a 63×1.4 NA objective and 405, 488 and 561 nm laser lines. For both co-localization and photo-depletion experiments, single image planes with an optical section thickness of 0.7 um were acquired. In order to exclude spurious co-localization by cross-excitation or bleed-through, only a narrow band of emission fluorescence was detected (GFP: Excitation 488 nm, Emission 493–532 nm, mCherry: Excitation 561 nm, Emission 599–708 nm). Using those settings, we did not observe any bleed-through or cross-excitation, even when observing extremely bright samples. To deplete the freely diffusible cytoplasmic pool of the respective GFP fusions, a small region of interest (ROI) was repeatedly bleached using 405 nm and 488 nm laser lines. Loss of fluorescence was monitored using a second ROI at the opposite end of the observed cell. During bleaching, only these two areas of the cell were recorded to minimize photobleaching of the rest of the cell. After complete depletion of the cytoplasmic GFP-fusion pool, as estimated by the lack of further photobleaching in the second ROI, an image of the whole cell was acquired.

Image processing and analysis was performed using ImageJ. Only cropping and linear adjustments of brightness and contrasts were performed. In order to visualize the weak signals after photobleaching, a different scaling of the display range in comparison to the non-bleached images was used; also this adjustment was linear.

### Computational analysis

For the identification of C-terminal protein extensions containing a peroxisomal targeting signal the TransTerm [63] database was used to retrieve 3′ flanking sequences from several organisms. The sequences were processed using regular expressions to retain only sequences between the regular stop codon to the next in-frame stop codon. Sequences were translated using Virtual Ribosome [64]. C-terminal tripeptides of the translated sequences were scanned for putative PTS1 motifs using the following regular expression:

(∧\*[∧*]*?([ASTPCE]RL|[SATPCVNG]KL|S[SNH]L|ARI|S[KR]M|[AG]NL|SN[IM]|[SA][KRQ]Y|HHL|[QS][KRQ]F)\*). PTS2 motifs were predicted with TargetSignalPredictor (http://www.peroxisomedb.org). Data analysis was performed with NCBI (National Center for Biotechnology Information) Basic local alignment search tool (Blast) [65], other NCBI resources and GenRE - MIPS Ustilago maydis DataBase.

## Supporting Information

Figure S1 The predicted PTS1 motifs in the C-terminal extensions of human MDH1 and LDHB are functional. The TGA stop codons of full-length *MDH1* and *LDHB* were mutated to TGG. Mutated variants were fused to GFP (GFP-MDH1_pex_ and GFP-LDHB_pex_, respectively) and analyzed by fluorescence microscopy before and after repeated photobleaching (upper panel). The PTS1 motifs of human LDHB_pex_ or MDH1_pex_ were fused to GFP and analyzed for peroxisomal localization in HeLa cells (lower panel). Scale bars represent 10 µm.
(PDF)

Figure S2 Analysis of peroxisomal targeting of MDH1 via translational readthrough in HeLa cells. GFP-MDH1 and GFP-MDH1ΔPTS1 were expressed in HeLa cells together with the peroxisomal marker mCherry-SKL. Intracellular localization was followed by fluorescence microscopy before and after repeated photobleaching. Magnified areas are indicated and shown below the micrographs. Scale bars represent 10 µm.
(PDF)

Figure S3 Peroxisomal targeting of NADP-dependent isocitrate dehydrogenase via readthrough. (A) 3′ sequence of the *D. melanogaster idh* gene and its translation. The readthrough element and the PTS1 are highlighted. (B) Different mechanisms leading to peroxisomal targeting of Idh homologs are shown. In some organisms the PTS1 is part of the original open reading frame indicated by no readthr. and …NNN*. (C). The 12 C-terminal amino acids of *U. maydis* Idp1 were fused to GFP and co-expressed with mCherry-SKL in *U. maydis* cells. Scale bars represent 10 µm.
(PDF)

Table S1 Prediction of readthrough derived PTS1 motifs for all *U. maydis* genes ending on TGA CT.
(DOCX)

Table S2 Phylogenetic conservation of TGA CT containing genes with PTS1 encoding C-terminal extensions.
(DOCX)

Table S3 *U. maydis* strains used in this study.
(DOCX)

Table S4 Oligonucleotides and plasmids.
(DOCX)
