# Supplementary figures and images for: Ribosomal Readthrough at a Short UGA Stop Codon Context Triggers Dual Localization of Metabolic Enzymes in Fungi and Animals
Source: PLoS Genet. 2014 Oct 23;10(10):e1004685. doi: 10.1371/journal.pgen.1004685 (PMC4207609; doi:10.1371/journal.pgen.1004685)

Supplementary Figure S1

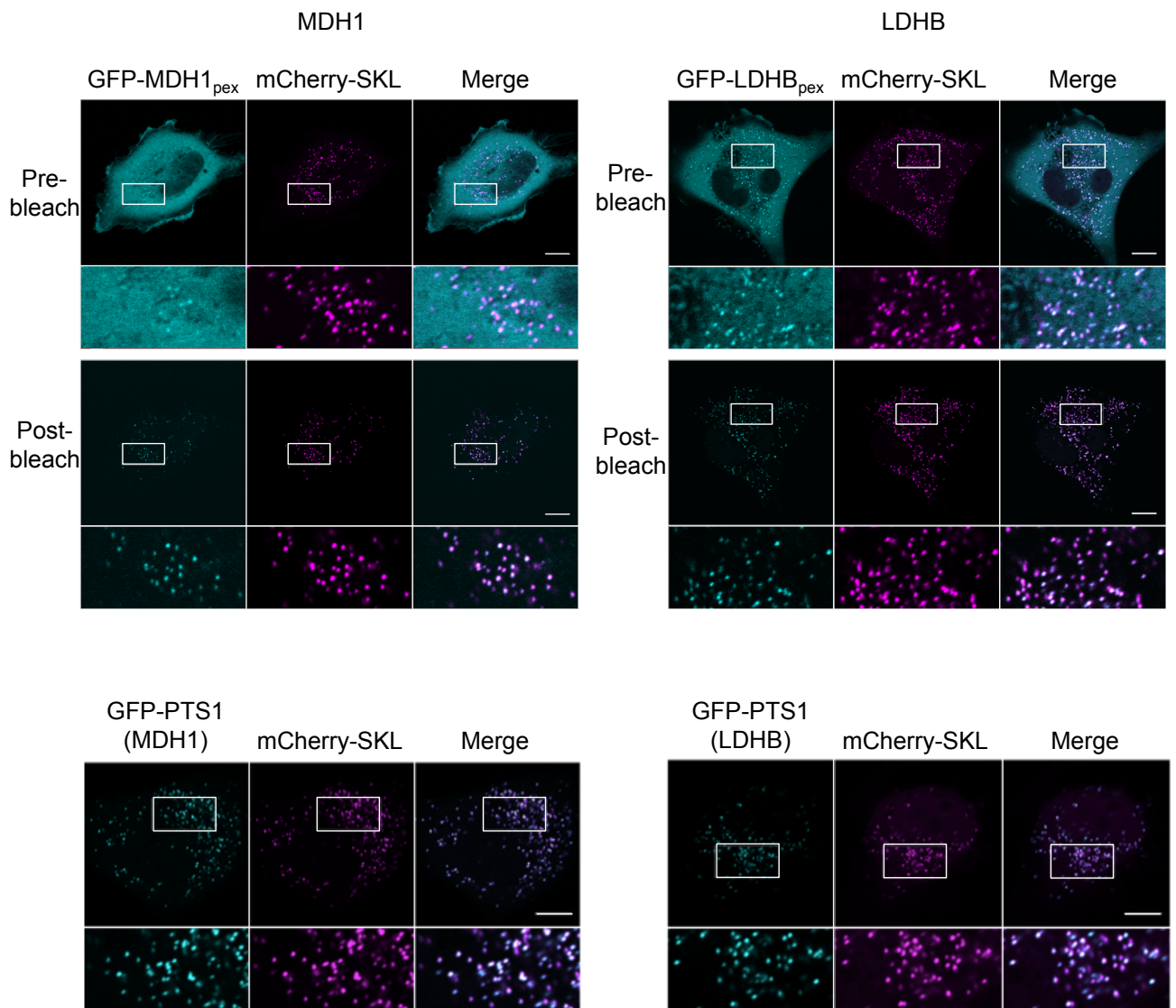

Supplement: Figure S1 — The predicted PTS1 motifs in the C-terminal extensions of human MDH1 and LDHB are functional. The TGA stop codons of full-length MDH1 and LDHB were mutated to TGG. Mutated variants were fused to GFP (GFP-MDH1pex and GFP-LDHBpex, respectively) and analyzed by fluorescence microscopy before and after repeated photobleaching (upper panel). The PTS1 motifs of human LDHBpex or MDH1pex were fused to GFP and analyzed for peroxisomal localization in HeLa cells (lower panel). Scale bars represent 10 µm. (PDF) [file pgen.1004685.s001.pdf]

Supplementary Figure S2

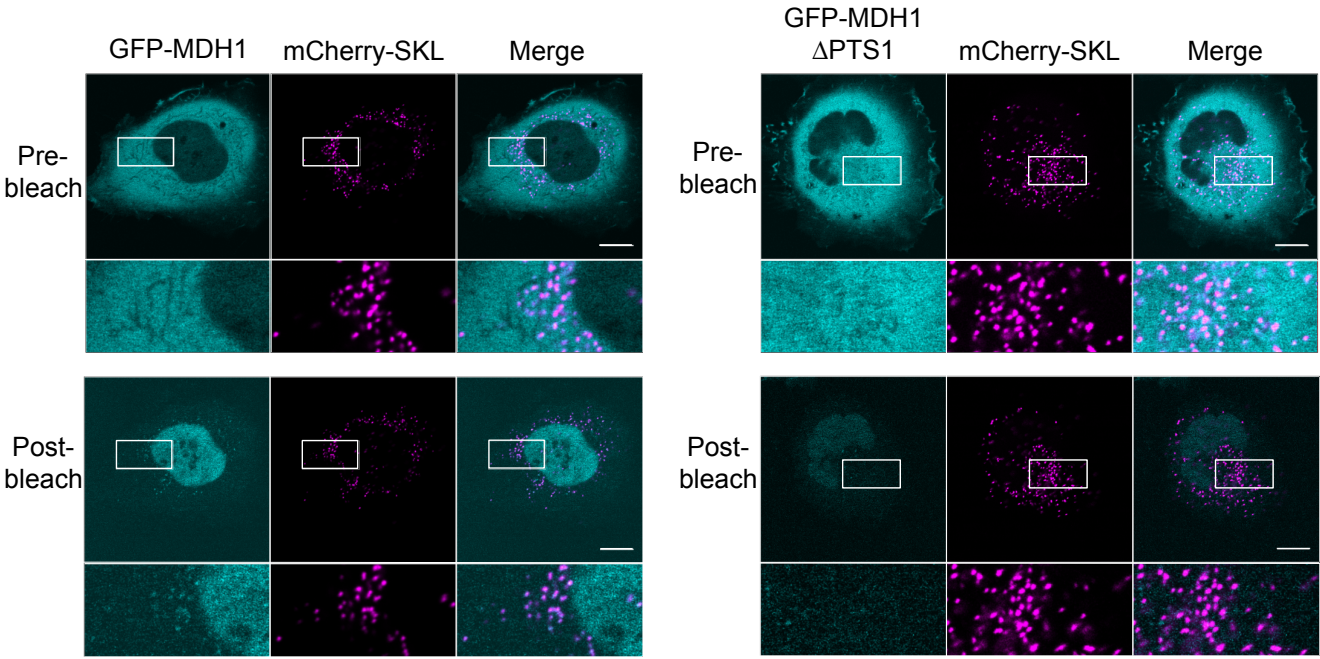

Supplement: Figure S2 — Analysis of peroxisomal targeting of MDH1 via translational readthrough in HeLa cells. GFP-MDH1 and GFP-MDH1ΔPTS1 were expressed in HeLa cells together with the peroxisomal marker mCherry-SKL. Intracellular localization was followed by fluorescence microscopy before and after repeated photobleaching. Magnified areas are indicated and shown below the micrographs. Scale bars represent 10 µm. (PDF) [file pgen.1004685.s002.pdf]
