# Supplementary material for: Ribosomal Readthrough at a Short UGA Stop Codon Context Triggers Dual Localization of Metabolic Enzymes in Fungi and Animals
Source: PLoS Genet. 2014 Oct 23;10(10):e1004685. doi: 10.1371/journal.pgen.1004685 (PMC4207609; doi:10.1371/journal.pgen.1004685)

Supplementary Figure S3

A

D. melanogaster NADP-dependent isocitrate dehydrogenase (*Idh*; Gene ID: 44291)

```
AAA TGA CTA TCC GGC ACT CAG TCC GAG CAG CAG GCC TCG CAT CTC TAG
 K   *   L   S   G   T   Q   S   E   Q   Q   A   S   H   L   *
```

B

	Readthrough context	C-terminal extension	PTS1 score
Drosophila melanogaster	TGACTA	*N$_{10}$SHL*	+2.0
Apis mellifera	TGACTA	*N$_{9}$SKY*	+1.6
Tribolium castaneum	TGACTA	*N$_{3}$ARL*	+11.2
Caenorhabditis elegans	no readthr.	...QAH*	-59.2
Aplysia californica	TGACTA	*N$_{16}$SKL*	+9.7
Xenopus laevis	no readthr.	...LKL*	-9.2
Gallus gallus	no readthr.	...PKL*	+1.3
Homo sapiens	no readthr.	...AKL*	+8.5
Ustilago maydis	no readthr.	...GKL*	+1.1

insects
birds
nematodes
mammals
molluscs
fungi
amphibians

C

U. maydis Idp1; Um06111

BF | GFP-PTS1$_{IDP1}$ | mCherry-SKL | Merge

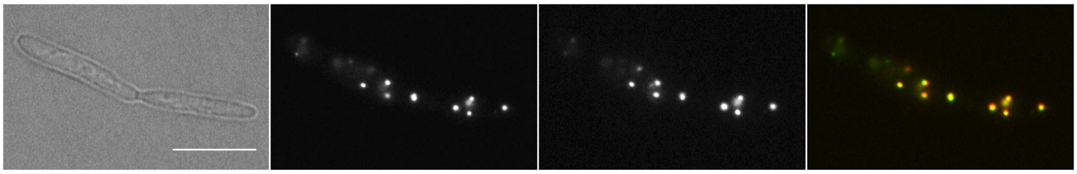

Supplement: Figure S3 — Peroxisomal targeting of NADP-dependent isocitrate dehydrogenase via readthrough. (A) 3′ sequence of the D. melanogaster idh gene and its translation. The readthrough element and the PTS1 are highlighted. (B) Different mechanisms leading to peroxisomal targeting of Idh homologs are shown. In some organisms the PTS1 is part of the original open reading frame indicated by no readthr. and …NNN*. (C). The 12 C-terminal amino acids of U. maydis Idp1 were fused to GFP and co-expressed with mCherry-SKL in U. maydis cells. Scale bars represent 10 µm. (PDF) [file pgen.1004685.s003.pdf]
