# Supplementary material for: Ribosomal Readthrough at a Short UGA Stop Codon Context Triggers Dual Localization of Metabolic Enzymes in Fungi and Animals
Source: PLoS Genet. 2014 Oct 23;10(10):e1004685. doi: 10.1371/journal.pgen.1004685 (PMC4207609; doi:10.1371/journal.pgen.1004685)
Supplement: Table S1 — Prediction of readthrough derived PTS1 motifs for all U. maydis genes ending on TGA CT. (DOCX) [file pgen.1004685.s004.docx]

| **Table S1:** Prediction of readthrough derived PTS1 motifs for all *U. maydis* genes ending on TGA CT | | | | | |
| --- | --- | --- | --- | --- | --- |
| MUMDBAccession | Annotation | Readthrough context | PTS1 | PTS1 score | Classification |
| um04797 | probable RPE1- D-ribulose-5-phosphate-3-epimerase | TGA CTT | VEGKLARRPAKL* | 12.747 | Targeted |
| um04871 | probable PGK1- phosphoglycerate kinase | TGA CTC | SEKSLDAAVPKL* | 9.387 | Targeted |
| um03229 | Related to triosephosphate isomerase | TGA CTA | ANA*LAGQSARI* | 0.734 | Targeted |
| um00254 | uncharacterized protein | TGA CTC | IRD*LTLRIFKL* | -2.905 | Twilight zone |
| um05126 | related to NCL1- tRNA-methyltransferase | TGA CTG | *LIHVRALAPHL* | -3.345 | Twilight zone |
| um12293 | related to PPM1- carboxyl methyltransferase | TGA CTC | PPIGRKNSMASF* | -5.818 | Twilight zone |
| um10747 | related to Exosome complex exonuclease RRP43 | TGA CTT | SNSPSTQHSNLM* | -9.282 | Twilight zone |
| um03211 | related to peroxiredoxin q | TGA CTC | GHANIATHFCNV* | -9.950 | Twilight zone |
| um01568 | uncharacterized protein | TGA CTC | LQLGVSAIRLRY* | -12.896 | Not targeted |
| um02690 | uncharacterized protein | TGA CTT | RTTISATNRR*L* | -15.433 | Not targeted |
| um03914 | related to Lactaldehyde reductase | TGA CTA | DSVYQSLAHERL* | -16.694 | Not targeted |
| um03655 | related to VTC1 subunit of the vacuolar transporter chaperone complex | TGA CTG | PQRNKVQLARQL* | -17.130 | Not targeted |
| um04703 | probable phosphomannomutase | TGA CTC | ELFDLA*LAARH* | -20.259 | Not targeted |
| um01841 | uncharacterized protein | TGA CTC | QSRLSRRNQRKV* | -20.311 | Not targeted |
| um05608 | related to U1 small nuclear ribonucleoprotein C | TGA CTT | RIKRSLAFDARQ* | -22.241 | Not targeted |
| um11060 | uncharacterized protein | TGA CTT | LVNSWSFMLPRT* | -24.626 | Not targeted |
| um02653 | related to alpha-1,3-mannosyltransferase alg2 | TGA CTG | VAQAPLPTPL*L* | -24.869 | Not targeted |
| um01584 | uncharacterized protein | TGA CTC | HRPHHSAIQYSR* | -26.434 | Not targeted |
| um06513 | uncharacterized protein | TGA CTT | NPDHP*LRSVHR* | -26.645 | Not targeted |
| um04650 | uncharacterized protein | TGA CTG | ATASNGIEQTSK* | -27.727 | Not targeted |
| um05095 | uncharacterized protein | TGA CTC | DS*LVTCRSLRS* | -28.340 | Not targeted |
| um01795 | uncharacterized protein | TGA CTG | HPCIIVKFINRF* | -28.595 | Not targeted |
| um02538 | uncharacterized protein | TGA CTA | LRSTEFQFGSTG* | -28.661 | Not targeted |
| um02374 | high-affinity sucrose transporter | TGA CTT | SLAATYFGSMNV* | -29.699 | Not targeted |
| um00280 | related to Glutaminyl-peptide cyclotransferase precursor | TGA CTT | RRELDKMRVGSK* | -30.880 | Not targeted |
| um02655 | related to RNA binding motif protein | TGA CTG | SIRSSGGIHQSS* | -31.067 | Not targeted |
| um01041 | uncharacterized protein | TGA CTA | NPLIRRGS*LLI* | -31.082 | Not targeted |
| um01772 | related to Aldose 1-epimerase precursor | TGA CTG | MSLDNASQSKSS* | -33.258 | Not targeted |
| um03517 | uncharacterized protein | TGA CTT | YSSRGQCALNAA* | -33.820 | Not targeted |
| um03570 | uncharacterized protein | TGA CTG | PSSFSSTR*LGF* | -34.009 | Not targeted |
| um04160 | Dkh6, novel virulence factor | TGA CTT | SQSLRFLHSSVR* | -34.295 | Not targeted |
| um11318 | probable N-terminal acetyltransferase complex subunit ARD1 | TGA CTT | ITSVHAKSKGLD* | -34.834 | Not targeted |
| um02722 | uncharacterized protein | TGA CTT | NGKNPPKHGWLS* | -34.929 | Not targeted |
| um06141 | uncharacterized protein | TGA CTG | HH*LVHSRINDV* | -35.188 | Not targeted |
| um00589 | related to GLO4-glyoxalase II | TGA CTG | NI*LRLNSFNLS* | -36.538 | Not targeted |
| um00997 | related to GCD7- translation initiation factor eIF2b | TGA CTC | *LSSNTRSTDRR* | -36.670 | Not targeted |
| um01307 | uncharacterized protein | TGA CTC | RRNENRSNLHAC* | -37.356 | Not targeted |
| um02112 | uncharacterized protein | TGA CTC | CGRVTSRGRDQS* | -37.418 | Not targeted |
| um06474 | uncharacterized protein | TGA CTC | LSP*LSIPFDSP* | -38.074 | Not targeted |
| um02451 | uncharacterized protein | TGA CTC | VQLFFRQPRRDL* | -38.617 | Not targeted |
| um02859 | probable CRM1-nuclear export factor, exportin | TGA CTC | DEEL*LIRELLY* | -39.805 | Not targeted |
| um03147 | related to Adapter-related protein complex 3 delta 1 subunit | TGA CTT | MCKRLCQHGESA* | -40.765 | Not targeted |
| um01834 | uncharacterized protein | TGA CTC | R*LWLIELIPPL* | -41.649 | Not targeted |
| um00758 | uncharacterized protein | TGA CTC | QHSVPIHAPLTT* | -42.210 | Not targeted |
| um06389 | uncharacterized protein | TGA CTC | LHCVWNKHKVTK* | -42.977 | Not targeted |
| um01152 | related to PSY2- subunit of protein phosphatase PP4 complex | TGA CTG | RIDVSSFIPTLS* | -43.775 | Not targeted |
| um02619 | related to TAF2- component of TFIID complex | TGA CTG | LQCLCDHSHMCL* | -45.078 | Not targeted |
| um11967 | uncharacterized protein | TGA CTG | SQNA*LADWLTG* | -45.484 | Not targeted |
| um11481 | related to aquaporin | TGA CTC | VRPSPHDALYPP* | -45.960 | Not targeted |
| um02308 | uncharacterized protein | TGA CTC | TSPFHTMTMKLR* | -46.499 | Not targeted |
| um12018 | uncharacterized protein | TGA CTG | GHTCIPSFRRVR* | -46.926 | Not targeted |
| um05912 | probable DNA repair endonuclease rad2 | TGA CTC | PLTRPIKGDACD* | -46.982 | Not targeted |
| um00048 | uncharacterized protein | TGA CTG | *LYRSGCSVTFD* | -47.517 | Not targeted |
| um03540 | uncharacterized protein | TGA CTC | SRLLIRFVMHHA* | -49.104 | Not targeted |
| um00473 | uncharacterized protein | TGA CTG | AKGEGRRDCRDI* | -49.732 | Not targeted |
| um01511 | uncharacterized protein | TGA CTC | VTDHRCTGSRPG* | -51.947 | Not targeted |
| um02124 | related to ERG6-Delta(24)-sterol C-methyltransferase | TGA CTT | HKPEQAKGV*LY* | -53.882 | Not targeted |
| um01661 | uncharacterized protein | TGA CTT | SGSASAAVHVPR* | -53.897 | Not targeted |
| um03027 | related to MON1-required for fusion of cvt-vesicles and autophagosomes with the vacuole | TGA CTT | FLVNAGSF*LVP* | -55.563 | Not targeted |
| um00270 | uncharacterized protein | TGA CTC | RHVHVNAE*LDA* | -57.280 | Not targeted |
| um04120 | uncharacterized protein | TGA CTG | IEAVSVAPFRAR* | -58.067 | Not targeted |
| um03902 | related to 5-oxoprolinase | TGA CTT | QCIHECFVMRDV* | -61.666 | Not targeted |
| um03612 | uncharacterized protein | TGA CTG | TSLFSSIVNRES* | -66.766 | Not targeted |
| um06439 | uncharacterized protein | TGA CTT | RRDRCLHFFLPS* | -68.117 | Not targeted |
| um10368 | related to Heat shock factor protein | TGA CTG | LLFTHAWWLLFV* | -81.101 | Not targeted |
| um03456 | uncharacterized protein | TGA CTT | LVHPTLFLHRIA* | -88.203 | Not targeted |

| **Supplementary Table S2:** Phylogenetic conservation of TGA CT containing genes with PTS1 encoding C-terminal extensions | | | | | | |
| --- | --- | --- | --- | --- | --- | --- |
| Organism | Accession | Annotation | Readthrough context | PTS1 | PTS1 Score | Classification |
| *Homo sapiens* | XM_005264320 | Malate dehydrogenase 1, NAD | TGA CTA | KCFKAEESKCRL* | 4.925 | Targeted |
| *Mus musculus* | NM_008618 | Malate dehydrogenase 1, NAD | TGA CTA | RQPKAEESKCRL* | 5.118 | Targeted |
| *Gallus gallus* | NM_001006395 | Malate dehydrogenase 1, NAD | TGA CTA | SHLRVEESKSRL* | 4.662 | Targeted |
| *Xenopus laevis* | NM_001089866 | Malate dehydrogenase 1, NAD | TGA CTA | MHLTPEKMKSSL* | 7.086 | Targeted |
| *Caenorhabditis elegans* | NM_072255 | Malate dehydrogenase 1, NAD | No readthrough | DDALKACDDANI* | -20.394 | Not targeted |
| *Drosophila melanogaster* | NP_609394 | Malate dehydrogenase 1, NAD | No readthrough | ALSVLDSNVSNL* | 1.101 | Targeted |
|  |  |  |  |  |  |  |
| *Homo sapiens* | Y00711 | Lactate dehydrogenase B | TGA CTA | DLKDL*LVSSRL* | 5.331 | Targeted |
| *Mus musculus* | NM_008492 | Lactate dehydrogenase B | TGA CTG | DLKDL*LPVSRL* | 8.648 | Targeted |
|  |  |  |  |  |  |  |
| *Homo sapiens* | NM_006903 | Inorganic pyrophosphatase | TGA TTG | KHLKFCCQDSHL* | -6.288 | Twilight zone |
| *Mus musculus* | NM_026438 | Inorganic pyrophosphatase | TGA AGA | PGSIRCKRFSKL* | 12.147 | Targeted |
| *Gallus gallus* | XM_001232699 | Inorganic pyrophosphatase | TGA GGG | MSGLVGARRHHL* | 7.795 | Targeted |
| *Caenorhabditis elegans* | CELE_C47E12.4 | Inorganic pyrophosphatase | TGA CTA | VRPGSREASSKL* | 13.726 | Targeted |
| *Drosophila melanogaster* | NM_001259565 | Inorganic pyrophosphatase (alternative transcript) | No readthrough | DTGKVHYIRSNL* | 9.880 | Targeted |
|  |  |  |  |  |  |  |
| *Homo sapiens* | NM_153498 | calcium/calmodulin-dependent protein kinase ID | TGA CTG | GSGAVYTNLAKL* | 4.597 | Targeted |
|  |  |  |  |  |  |  |
| *Homo sapiens* | XM_005267198 | synaptojanin 2 (SYNJ2) | TGA CTG | GCSPIECIPSSL* | 3.711 | Targeted |
|  |  |  |  |  |  |  |
| *Homo sapiens* | BC157827 | membrane bound O-acyltransferase domain containing 2 | TGA TCG | RHSSLTQ*SGRL* | 4.210 | Targeted |
| *Mus musculus* | BC025020 | membrane bound O-acyltransferase domain containing 2 | TGA CTG | *LRERVMAVSRL* | 7.317 | Targeted |
|  |  |  |  |  |  |  |
| *Caenorhabditis elegans* | X77020 | Zinc/copper superoxide dismutase | TGA CTA | LAAPQ*LPESRL* | -2.218 | Twilight zone |
|  |  |  |  |  |  |  |
| *Drosophila melanogaster* | NM_168267 | Isocitrate dehydrogenase (Idh) | TGA CTA | SGTQSEQQASHL* | 2.010 | Targeted |

| **Supplementary Table S3:** *U. maydis* strains used in this study | | | |
| --- | --- | --- | --- |
| Strain | Genotype | Resistance | Reference |
| Bub8 | *a2 b4* | - | (*31*) |
| Bub8 mCherry-SKL | *a2 b4 P_otef_:mcherry-SKL* | Hyg^R^ | (*10*) |
| Bub8 TPI-GFP | *a2 b4 ip^R^[P_otef_:tpi-egfp]ip^S^* | Cbx^R^ | This study |
| Bub8 TPI+GFP | *a2 b4 ip^R^[P_otef_:tpi+egfp]ip^S^* | Cbx^R^ | This study |
| Bub8 TPI+3-GFP | *a2 b4 ip^R^[P_otef_:tpi+3-egfp]ip^S^* | Cbx^R^ | This study |
| Bub8 TPI+9-GFP | *a2 b4 ip^R^[P_otef_:tpi+9-egfp]ip^S^* | Cbx^R^ | This study |
| Bub8 TPI+24-GFP | *a2 b4 ip^R^[P_otef_:tpi+24-egfp]ip^S^* | Cbx^R^ | This study |
| Bub8 mCherry-TAACTA-GFP | *a2 b4 ip^R^[P_otef_:mcherry-TAACTA-egfp]ip^S^* | Cbx^R^ | This study |
| Bub8 mCherry-TAGCTA-GFP | *a2 b4 ip^R^[P_otef_:mcherry-TAGCTA-egfp]ip^S^* | Cbx^R^ | This study |
| Bub8 mCherry-TGACTA-GFP | *a2 b4 ip^R^[P_otef_:mcherry-TGACTA-egfp]ip^S^* | Cbx^R^ | This study |
| Bub8 mCherry-TGACTC-GFP | *a2 b4 ip^R^[P_otef_:mcherry-TGACTC-egfp]ip^S^* | Cbx^R^ | This study |
| Bub8 mCherry-TGACTG-GFP | *a2 b4 ip^R^[P_otef_:mcherry-TGACTG-egfp]ip^S^* | Cbx^R^ | This study |
| Bub8 mCherry-TGACTT-GFP | *a2 b4 ip^R^[P_otef_:mcherry-TGACTT-egfp]ip^S^* | Cbx^R^ | This study |
| Bub8 mCherry-TGACAA-GFP | *a2 b4 ip^R^[P_otef_:mcherry-TGACAA-egfp]ip^S^* | Cbx^R^ | This study |
| Bub8 mCherry-TGACCA-GFP | *a2 b4 ip^R^[P_otef_:mcherry-TGACCA-egfp]ip^S^* | Cbx^R^ | This study |
| Bub8 mCherry-TGACGA-GFP | *a2 b4 ip^R^[P_otef_:mcherry-TGACGA-egfp]ip^S^* | Cbx^R^ | This study |
| Bub8 mCherry-TGAATA-GFP | *a2 b4 ip^R^[P_otef_:mcherry-TGAATA-egfp]ip^S^* | Cbx^R^ | This study |
| Bub8 mCherry-TGAGTA-GFP | *a2 b4 ip^R^[P_otef_:mcherry-TGAGTA-egfp]ip^S^* | Cbx^R^ | This study |
| Bub8 mCherry-TGATTA-GFP | *a2 b4 ip^R^[P_otef_:mcherry-TGATTA-egfp]ip^S^* | Cbx^R^ | This study |
| Bub8 mCherry-SKL GFP-PTS1 (Art1) | *a2 b4 P_otef_:mcherry-SKL ip^R^[P_otef_:egfp- PTS1(Art1)]ip^S^* | Hyg^R^ ,Cbx^R^ | This study |
| Bub8 mCherry-SKL GFP-PTS1 (Rpe1) | *a2 b4 P_otef_:mcherry-SKL ip^R^[P_otef_: egfp- PTS1(Rpe1)]ip^S^* | Hyg^R^ ,Cbx^R^ | This study |
| Bub8 mCherry-SKL GFP-PTS1 (Idp1) | *a2 b4 P_otef_:mcherry-SKL ip^R^[P_otef_: egfp- PTS1(Idp1)]ip^S^* | Hyg^R^ ,Cbx^R^ | This study |

**Supplementary Table S4:** Oligonucleotides and plasmids

| Plasmid | | Oligonucleotides | | Sequence | Restriction site(s) | Vector |
| --- | --- | --- | --- | --- | --- | --- |
| pTPI-GFP | | TPI fwd | | ctgaggatccgatggctcgcactttcttcgtcggtg | BamHI | potef-GFP-Ala_6_-MMXN |
|  |  | TPI-GFP rev | | ctgaccatggcccaagcgttagcgttgacgatatcg | NcoI |  |
| pTPI+GFP | | TPI fwd | | ctgaggatccgatggctcgcactttcttcgtcggtg | BamHI | potef-GFP-Ala_6_-MMXN |
|  |  | TPI+GFP rev | | ctgaccatggctcaagcgttagcgttgacgatatcg | NcoI |  |
| pTPI+3-GFP | | TPI fwd | | ctgaggatccgatggctcgcactttcttcgtcggtg | BamHI | potef-GFP-Ala_6_-MMXN |
|  |  | TPI+3-GFP rev | | ctgaccatggctagtcaagcgttagcgttgacgata | NcoI |  |
| pTPI+9-GFP | | TPI fwd (MG579) | | ctgaggatccgatggctcgcactttcttcgtcggtg | BamHI | potef-GFP-Ala_6_-MMXN |
|  |  | TPI+9-GFP rev | | ctgaccatggcgccagctagtcaagcgttagcgttgacga | NcoI |  |
| pTPI+24-GFP | | TPI fwd | | ctgaggatccgatggctcgcactttcttcgtcggtg | BamHI | potef-GFP-Ala_6_-MMXN |
|  |  | TPI+24-GFP rev | | ctgaccatggcgatcctagccgactggccagctagt | NcoI |  |
| pmCherry-TAACTA-GFP | | TAACTA fwd | | ctgaacgcgtgtaactaatggtgagcaagggcgaggagctgt | MluI | potef-mCherry-Ala_6_-MMXN |
|  |  | GFP rev | | gatctctagactacttgtacagctcgtccatgccgaga | Xbal |  |
| pmCherry-TAGCTA-GFP | | TAGCTA fwd | | ctgaacgcgtgtagctaatggtgagcaagggcgaggagctgt | MluI | potef-mCherry-Ala_6_-MMXN |
|  |  | GFP rev | | gatctctagactacttgtacagctcgtccatgccgaga | Xbal |  |
| pmCherry-TGACTA-GFP | | TGACTA fwd | | ctgaacgcgtgtgactaatggtgagcaagggcgaggagctgt | MluI | potef-mCherry-Ala_6_-MMXN |
|  |  | GFP rev | | gatctctagactacttgtacagctcgtccatgccgaga | Xbal |  |
| pmCherry-TGACTC-GFP | | TGACTC fwd | | ctgaacgcgtgtgactcatggtgagcaagggcgaggagctgt | MluI | potef-mCherry-Ala_6_-MMXN |
|  |  | GFP rev | | gatctctagactacttgtacagctcgtccatgccgaga | Xbal |  |
| pmCherry-TGACTG-GFP | | TGACTG fwd | | ctgaacgcgtgtgactgatggtgagcaagggcgaggagctgt | MluI | potef-mCherry-Ala_6_-MMXN |
|  |  | GFP rev | | gatctctagactacttgtacagctcgtccatgccgaga | Xbal |  |
| pmCherry-TGACTT-GFP | | TGACTT fwd | | ctgaacgcgtgtgacttatggtgagcaagggcgaggagctgt | MluI | potef-mCherry-Ala_6_-MMXN |
|  |  | GFP rev | | gatctctagactacttgtacagctcgtccatgccgaga | Xbal |  |
| pmCherry-TGACAA-GFP | | TGACAA fwd | | ctgaacgcgtgtgacaaatggtgagcaagggcgaggagctgt | MluI | potef-mCherry-Ala_6_-MMXN |
|  |  | GFP rev | | gatctctagactacttgtacagctcgtccatgccgaga | Xbal |  |
| pmCherry-TGACCA-GFP | | TGACCA fwd | | ctgaacgcgtgtgaccaatggtgagcaagggcgaggagctgt | MluI | potef-mCherry-Ala_6_-MMXN |
|  |  | GFP rev | | gatctctagactacttgtacagctcgtccatgccgaga | Xbal |  |
| pmCherry-TGACGA-GFP | | TGACGA fwd | | ctgaacgcgtgtgacgaatggtgagcaagggcgaggagctgt | MluI | potef-mCherry-Ala_6_-MMXN |
|  |  | GFP rev | | gatctctagactacttgtacagctcgtccatgccgaga | Xbal |  |
| pmCherry-TGAATA-GFP | | TGAATA fwd | | ctgaacgcgtgtgaataatggtgagcaagggcgaggagctgt | MluI | potef-mCherry-Ala_6_-MMXN |
|  |  | GFP rev | | gatctctagactacttgtacagctcgtccatgccgaga | Xbal |  |
| pmCherry-TGAGTA-GFP | | TGAGTA fwd | | ctgaacgcgtgtgagtaatggtgagcaagggcgaggagctgt | MluI | potef-mCherry-Ala_6_-MMXN |
|  |  | GFP rev | | gatctctagactacttgtacagctcgtccatgccgaga | Xbal |  |
| pmCherry-TGATTA-GFP | | TGATTA fwd | | ctgaacgcgtgtgattaatggtgagcaagggcgaggagctgt | MluI | potef-mCherry-Ala_6_-MMXN |
|  |  | GFP rev | | gatctctagactacttgtacagctcgtccatgccgaga | Xbal |  |
| pGFP-PTS1 (Art1) | | PTS1 (Art1) 5’ | | cgcgtgaacggcgtcaaagcaatcctcgacagtgtgtatcagtcactagcgcacgagcggctgtaat | MluI, XbaI | potef-GFP-Ala_6_-MMXN |
|  |  | PTS1 (Art1) 3’ | | ctagattacagccgctcgtgcgctagtgactgatacacactgtcgaggattgctttgacgccgttca |  |  |
| pGFP-PTS1 (Rpe1) | | PTS1 (Rpe1) 5’ | | cgcgtgagtatcacgcctgctgccagtgcggtcgagggtaaatcacttgcgagacggccggctaagctttgat | MluI, XbaI | potef-GFP-Ala_6_-MMXN |
|  |  | PTS1 (Rpe1) 3’ | | ctagatcaaagcttagccggccgtctcgcaagtgatttaccctcgaccgcactggcagcaggcgtgatactca |  |  |
| pGFP-PTS1 (Idp1) | | PTS1 (Idp1) 5’ | | cgcgtaaagctccagactcgcggcatcgaggctggcaagctttgat | MluI, XbaI | potef-GFP-Ala_6_-MMXN |
|  |  | PTS1 (Idp1) 3’ | | ctagatcaaagcttgccagcctcgatgccgcgagtctggagcttta |  |  |
| pHA-LDH+Myc | | HA-LDH fwd | | atatggatccatgtacccatacgatgttccagattacgcagcaactcttaaggaaaaactc | BamHI | pcDNA^TM^3.1 |
|  |  | LDH+Myc rev | | atatgaattcttacaggtcctcctcgctaatgagtttctgctctcacaggtcttttaggtccttc | EcoRI |  |
| pHA-LDH-Myc | | HA-LDH fwd | | atatggatccatgtacccatacgatgttccagattacgcagcaactcttaaggaaaaactc | BamHI | pcDNA^TM^3.1 |
|  |  | LDH-Myc rev | | atatgaattcttacaggtcctcctcgctaatgagtttctgctccaggtcttttaggtccttctgg | EcoRI |  |
| pHA-LDH-TGACTA-Myc | | HA-LDH fwd | | atatggatccatgtacccatacgatgttccagattacgcagcaactcttaaggaaaaactc | BamHI | pcDNA^TM^3.1 |
|  |  | LDH+TGACTA-Myc rev | | atatgaattcttacaggtcctcctcgctaatgagtttctgctctagtcacaggtcttttaggtcc | EcoRI |  |
| pHA-LDH-TGA-18-Myc | | HA-LDH fwd | | atatggatccatgtacccatacgatgttccagattacgcagcaactcttaaggaaaaactc | BamHI | pcDNA^TM^3.1 |
|  |  | LDH-TGA-18-Myc rev | | atatgaattcttacaggtcctcctcgctaatgagtttctgctccagcctagagctcactagtca | EcoRI |  |
| pHA-LDH-TAACTA-Myc | | HA-LDH fwd | | atatggatccatgtacccatacgatgttccagattacgcagcaactcttaaggaaaaactc | BamHI | pcDNA^TM^3.1 |
|  |  | LDH-TAACTA-Myc rev | | atatgaattcttacaggtcctcctcgctaatgagtttctgctctagttacaggtcttttaggtcc |  |  |
| pHA-LDH-TAGCTA-Myc | | HA-LDH fwd | | atatggatccatgtacccatacgatgttccagattacgcagcaactcttaaggaaaaactc | BamHI | pcDNA^TM^3.1 |
|  |  | LDH-TAACTA-Myc rev | | atatgaattcttacaggtcctcctcgctaatgagtttctgctctagctacaggtcttttaggtcc |  |  |
| pGFP-TGACTA-Myc | | TGACTA-Myc 5‘ | | aattcaaaggacctaaaagacctgtgactagagcagaaactcattagcgaggaggacctgtaag | BamHI, EcoRI | pEGFP-C1 |
|  |  | TGACTA-Myc 3‘ | | gatccttacaggtcctcctcgctaatgagtttctgctctagtcacaggtcttttaggtcctttg |  |  |
| pGFP-TGACTT-Myc | | TGACTT-Myc 5‘ | | aattcaaaggacctaaaagacctgtgacttgagcagaaactcattagcgaggaggacctgtaag | BamHI, EcoRI | pEGFP-C1 |
|  |  | TGACTT-Myc 3‘ | | gatccttacaggtcctcctcgctaatgagtttctgctcaagtcacaggtcttttaggtcctttg |  |  |
| pGFP-TGACTC-Myc | | TGACTC-Myc 5‘ | | aattcaaaggacctaaaagacctgtgactcgagcagaaactcattagcgaggaggacctgtaag | BamHI, EcoRI | pEGFP-C1 |
|  |  | TGACTC-Myc 3‘ | | gatccttacaggtcctcctcgctaatgagtttctgctcgagtcacaggtcttttaggtcctttg |  |  |
| pGFP-TGACTG-Myc | | TGACTG-Myc 5‘ | | aattcaaaggacctaaaagacctgtgactggagcagaaactcattagcgaggaggacctgtaag | BamHI, EcoRI | pEGFP-C1 |
|  |  | TGACTG-Myc 3‘ | | gatccttacaggtcctcctcgctaatgagtttctgctccagtcacaggtcttttaggtcctttg |  |  |
| pGFP-TGACCA-Myc | | TGACCA-Myc 5‘ | | aattcaaaggacctaaaagacctgtgaccagagcagaaactcattagcgaggaggacctgtaag | BamHI, EcoRI | pEGFP-C1 |
|  |  | TGACCA-Myc 3‘ | | gatccttacaggtcctcctcgctaatgagtttctgctctggtcacaggtcttttaggtcctttg |  |  |
| pGFP-TGACAA-Myc | | TGACAA-Myc 5‘ | | aattcaaaggacctaaaagacctgtgacaagagcagaaactcattagcgaggaggacctgtaag | BamHI, EcoRI | pEGFP-C1 |
|  |  | TGACAA-Myc 3‘ | | gatccttacaggtcctcctcgctaatgagtttctgctcttgtcacaggtcttttaggtcctttg |  |  |
| pGFP-TGACGA-Myc | | TGACGA-Myc 5‘ | | aattcaaaggacctaaaagacctgtgacgagagcagaaactcattagcgaggaggacctgtaag | BamHI, EcoRI | pEGFP-C1 |
|  |  | TGACGA-Myc 3‘ | | gatccttacaggtcctcctcgctaatgagtttctgctctcgtcacaggtcttttaggtcctttg |  |  |
| pGFP-TGAATA-Myc | TGAATA-Myc 5‘ | | aattcaaaggacctaaaagacctgtgaatagagcagaaactcattagcgaggaggacctgtaag | | BamHI, EcoRI | pEGFP-C1 |
|  | TGA ATA-Myc 3‘ | | gatccttacaggtcctcctcgctaatgagtttctgctctattcacaggtcttttaggtcctttg | |  |  |
| pGFP-TGATTA-Myc | TGATTA-Myc 5‘ | | aattcaaaggacctaaaagacctgtgattagagcagaaactcattagcgaggaggacctgtaag | | BamHI, EcoRI | pEGFP-C1 |
|  | TGATTA-Myc 3‘ | | gatccttacaggtcctcctcgctaatgagtttctgctctaatcacaggtcttttaggtcctttg | |  |  |
| pGFP-TGAGTA-Myc | TGAGTA-Myc 5‘ | | aattcaaaggacctaaaagacctgtgagtagagcagaaactcattagcgaggaggacctgtaag | | BamHI, EcoRI | pEGFP-C1 |
|  | TGAGTA-Myc 3‘ | | gatccttacaggtcctcctcgctaatgagtttctgctctactcacaggtcttttaggtcctttg | |  |  |
| pmCherry-SKL | mCherry-SKL 5‘ | | gatccttacagcttggatagag | | BamHI, EcoRI | pmCherry-C1 |
|  | mCherry-SKL 3‘ | | aattctctatccaagctgtaag | |  |  |
| pEGFP-LDH | GFP-LDH fwd | | atatgaattccatggcaactcttaaggaaaaactc | | EcoRI | pEGFP-C1 |
|  | LDH rev | | atatggatcctcacaggtcttttaggtccttc | | BamHI |  |
| pEGFP-LdhB_Pex_ | GFP-LDH fwd | | atatgaattccatggcaactcttaaggaaaaactc | | EcoRI | pEGFP-C1 |
|  | LDH+PTS1 rev | | atatggatccaggctttgattctgtgagccc | | BamHI |  |
| pEGFP-LdhB_Cyt_ | GFP-LDH fwd | | atatgaattccatggcaactcttaaggaaaaactc | | EcoRI | pEGFP-C1 |
|  | LDH- PTS1 rev | | atatggatccctacagcctagagctcactagccacaggtcttttagg | | BamHI |  |
| pEGFP-Mdh1 | GFP-MDH fwd | | atatgaattccatgtctgaaccaatcagagtcc | | EcoRI | pEGFP-C1 |
|  | MDH rev | | atatggatcctcaggcagaggaaagaaattcaaaagc | | BamHI |  |
| pEGFP-Mdh1_Pex_ | GFP-MDH fwd | | atatgaattccatgtctgaaccaatcagagtcc | | EcoRI | pEGFP-C1 |
|  | MDH+PTS1 rev | | atatggatccactgtcattcacaaacctgtacc | | BamHI |  |
| pEGFP-Mdh1_Cyt_ | GFP-MDH fwd | | atatgaattccatgtctgaaccaatcagagtcc | | EcoRI | pEGFP-C1 |
|  | MDH-PTS1 rev | | atatggatcctcaaagacgacatttagattcttcagctttgaagcatttagtaacatcattgtctagccaggcagaggaaagaaattc | | BamHI |  |
| pEGFP-PTS1 (Mdh1) | PTS1-MDH-Sense | | aattctaaatgcttcaaagctgaagaatctaaatgtcgtctttgag | | EcoRI | pEGFP-C1 |
|  | PTS1-MDH-Antisense | | gatcctcaaagacgacatttagattcttcagctttgaagcatttag | | BamHI |  |
| pEGFP-PTS1 (LdhB) | PTS1-LDH-Sense | | aattctgacctaaaagacctgtggctagtgagctctaggctgtagg | | EcoRI | pEGFP-C1 |
|  | PTS1-LDH-Antisense | | gatccctacagcctagagctcactagccacaggtcttttaggtcag | | BamHI |  |
| pEGFP-PTS1 (Ppa2) | PTS1-PPA2-Sense | | aattctaaacatctgaaattctgctgtcaagattcccatctctaag | | EcoRI | pEGFP-C1 |
|  | PTS1-PPA2-Antisense | | gatccttagagatgggaatcttgacagcagaatttcagatgtttag | | BamHI |  |
