# Supplementary material for: Ribosomal Readthrough at a Short UGA Stop Codon Context Triggers Dual Localization of Metabolic Enzymes in Fungi and Animals
Source: PLoS Genet. 2014 Oct 23;10(10):e1004685. doi: 10.1371/journal.pgen.1004685 (PMC4207609; doi:10.1371/journal.pgen.1004685)
Supplement: Table S2 — Phylogenetic conservation of TGA CT containing genes with PTS1 encoding C-terminal extensions. (DOCX) [file pgen.1004685.s005.docx]

| **Table S2:** Phylogenetic conservation of TGA CT containing genes with PTS1 encoding C-terminal extensions | | | | | | |
| --- | --- | --- | --- | --- | --- | --- |
| Organism | Accession | Annotation | Readthrough context | PTS1 | PTS1 Score | Classification |
| *Homo sapiens* | XM_005264320 | Malate dehydrogenase 1 | TGA CTA | KCFKAEESKCRL* | 4.925 | Targeted |
| *Mus musculus* | NM_008618 | Malate dehydrogenase 1 | TGA CTA | RQPKAEESKCRL* | 5.118 | Targeted |
| *Gallus gallus* | NM_001006395 | Malate dehydrogenase 1 | TGA CTA | SHLRVEESKSRL* | 4.662 | Targeted |
| *Xenopus laevis* | NM_001089866 | Malate dehydrogenase 1 | TGA CTA | MHLTPEKMKSSL* | 7.086 | Targeted |
| *Caenorhabditis elegans* | NM_072255 | Malate dehydrogenase 1 | No readthrough | DDALKACDDANI* | -20.394 | Not targeted |
| *Drosophila melanogaster* | NP_609394 | Malate dehydrogenase 1 | No readthrough | ALSVLDSNVSNL* | 1.101 | Targeted |
|  |  |  |  |  |  |  |
| *Homo sapiens* | Y00711 | Lactate dehydrogenase B | TGA CTA | DLKDL*LVSSRL* | 5.331 | Targeted |
| *Mus musculus* | NM_008492 | Lactate dehydrogenase B | TGA CTG | DLKDL*LPVSRL* | 8.648 | Targeted |
|  |  |  |  |  |  |  |
| *Homo sapiens* | NM_006903 | Inorganic pyrophosphatase | TGA TTG | KHLKFCCQDSHL* | -6.288 | Twilight zone |
| *Mus musculus* | NM_026438 | Inorganic pyrophosphatase | TGA AGA | PGSIRCKRFSKL* | 12.147 | Targeted |
| *Gallus gallus* | XM_001232699 | Inorganic pyrophosphatase | TGA GGG | MSGLVGARRHHL* | 7.795 | Targeted |
| *Caenorhabditis elegans* | CELE_C47E12.4 | Inorganic pyrophosphatase | TGA CTA | VRPGSREASSKL* | 13.726 | Targeted |
| *Drosophila melanogaster* | NM_001259565 | Inorganic pyrophosphatase (alternative transcript) | No readthrough | DTGKVHYIRSNL* | 9.880 | Targeted |
|  |  |  |  |  |  |  |
| *Homo sapiens* | NM_153498 | calcium/calmodulin-dependent protein kinase ID | TGA CTG | GSGAVYTNLAKL* | 4.597 | Targeted |
|  |  |  |  |  |  |  |
| *Homo sapiens* | XM_005267198 | synaptojanin 2 (SYNJ2) | TGA CTG | GCSPIECIPSSL* | 3.711 | Targeted |
|  |  |  |  |  |  |  |
| *Homo sapiens* | BC157827 | membrane bound O-acyltransferase domain containing 2 | TGA TCG | RHSSLTQ*SGRL* | 4.210 | Targeted |
| *Mus musculus* | BC025020 | membrane bound O-acyltransferase domain containing 2 | TGA CTG | *LRERVMAVSRL* | 7.317 | Targeted |
|  |  |  |  |  |  |  |
| *Caenorhabditis elegans* | X77020 | Zinc/copper superoxide dismutase | TGA CTA | LAAPQ*LPESRL* | -2.218 | Twilight zone |
|  |  |  |  |  |  |  |
| *Drosophila melanogaster* | NM_168267 | Isocitrate dehydrogenase (Idh) | TGA CTA | SGTQSEQQASHL* | 2.010 | Targeted |
