# Supplementary material for: Ribosomal Readthrough at a Short UGA Stop Codon Context Triggers Dual Localization of Metabolic Enzymes in Fungi and Animals
Source: PLoS Genet. 2014 Oct 23;10(10):e1004685. doi: 10.1371/journal.pgen.1004685 (PMC4207609; doi:10.1371/journal.pgen.1004685)
Supplement: Table S3 — U. maydis strains used in this study. (DOCX) [file pgen.1004685.s006.docx]

| **Table S3:** *U. maydis* strains used in this study | | | |
| --- | --- | --- | --- |
| Strain | Genotype | Resistance | Reference |
| Bub8 | *a2 b4* | - | (*31*) |
| Bub8 mCherry-SKL | *a2 b4 P_otef_:mcherry-SKL* | Hyg^R^ | (*10*) |
| Bub8 TPI-GFP | *a2 b4 ip^R^[P_otef_:tpi-egfp]ip^S^* | Cbx^R^ | This study |
| Bub8 TPI+GFP | *a2 b4 ip^R^[P_otef_:tpi+egfp]ip^S^* | Cbx^R^ | This study |
| Bub8 TPI+3-GFP | *a2 b4 ip^R^[P_otef_:tpi+3-egfp]ip^S^* | Cbx^R^ | This study |
| Bub8 TPI+9-GFP | *a2 b4 ip^R^[P_otef_:tpi+9-egfp]ip^S^* | Cbx^R^ | This study |
| Bub8 TPI+24-GFP | *a2 b4 ip^R^[P_otef_:tpi+24-egfp]ip^S^* | Cbx^R^ | This study |
| Bub8 mCherry-TAACTA-GFP | *a2 b4 ip^R^[P_otef_:mcherry-TAACTA-egfp]ip^S^* | Cbx^R^ | This study |
| Bub8 mCherry-TAGCTA-GFP | *a2 b4 ip^R^[P_otef_:mcherry-TAGCTA-egfp]ip^S^* | Cbx^R^ | This study |
| Bub8 mCherry-TGACTA-GFP | *a2 b4 ip^R^[P_otef_:mcherry-TGACTA-egfp]ip^S^* | Cbx^R^ | This study |
| Bub8 mCherry-TGACTC-GFP | *a2 b4 ip^R^[P_otef_:mcherry-TGACTC-egfp]ip^S^* | Cbx^R^ | This study |
| Bub8 mCherry-TGACTG-GFP | *a2 b4 ip^R^[P_otef_:mcherry-TGACTG-egfp]ip^S^* | Cbx^R^ | This study |
| Bub8 mCherry-TGACTT-GFP | *a2 b4 ip^R^[P_otef_:mcherry-TGACTT-egfp]ip^S^* | Cbx^R^ | This study |
| Bub8 mCherry-TGACAA-GFP | *a2 b4 ip^R^[P_otef_:mcherry-TGACAA-egfp]ip^S^* | Cbx^R^ | This study |
| Bub8 mCherry-TGACCA-GFP | *a2 b4 ip^R^[P_otef_:mcherry-TGACCA-egfp]ip^S^* | Cbx^R^ | This study |
| Bub8 mCherry-TGACGA-GFP | *a2 b4 ip^R^[P_otef_:mcherry-TGACGA-egfp]ip^S^* | Cbx^R^ | This study |
| Bub8 mCherry-TGAATA-GFP | *a2 b4 ip^R^[P_otef_:mcherry-TGAATA-egfp]ip^S^* | Cbx^R^ | This study |
| Bub8 mCherry-TGAGTA-GFP | *a2 b4 ip^R^[P_otef_:mcherry-TGAGTA-egfp]ip^S^* | Cbx^R^ | This study |
| Bub8 mCherry-TGATTA-GFP | *a2 b4 ip^R^[P_otef_:mcherry-TGATTA-egfp]ip^S^* | Cbx^R^ | This study |
| Bub8 mCherry-SKL GFP-PTS1 (Art1) | *a2 b4 P_otef_:mcherry-SKL ip^R^[P_otef_:egfp- PTS1(Art1)]ip^S^* | Hyg^R^ ,Cbx^R^ | This study |
| Bub8 mCherry-SKL GFP-PTS1 (Rpe1) | *a2 b4 P_otef_:mcherry-SKL ip^R^[P_otef_: egfp- PTS1(Rpe1)]ip^S^* | Hyg^R^ ,Cbx^R^ | This study |
| Bub8 mCherry-SKL GFP-PTS1 (Idp1) | *a2 b4 P_otef_:mcherry-SKL ip^R^[P_otef_: egfp- PTS1(Idp1)]ip^S^* | Hyg^R^ ,Cbx^R^ | This study |
