# Supplementary material for: Ribosomal Readthrough at a Short UGA Stop Codon Context Triggers Dual Localization of Metabolic Enzymes in Fungi and Animals
Source: PLoS Genet. 2014 Oct 23;10(10):e1004685. doi: 10.1371/journal.pgen.1004685 (PMC4207609; doi:10.1371/journal.pgen.1004685)
Supplement: Table S4 — Oligonucleotides and plasmids. (DOCX) [file pgen.1004685.s007.docx]

**Table S4:** Oligonucleotides and plasmids

| Plasmid | | Oligonucleotides | | Sequence | Restriction site(s) | Vector |
| --- | --- | --- | --- | --- | --- | --- |
| pTPI-GFP | | TPI fwd | | ctgaggatccgatggctcgcactttcttcgtcggtg | BamHI | potef-GFP-Ala_6_-MMXN |
|  |  | TPI-GFP rev | | ctgaccatggcccaagcgttagcgttgacgatatcg | NcoI |  |
| pTPI+GFP | | TPI fwd | | ctgaggatccgatggctcgcactttcttcgtcggtg | BamHI | potef-GFP-Ala_6_-MMXN |
|  |  | TPI+GFP rev | | ctgaccatggctcaagcgttagcgttgacgatatcg | NcoI |  |
| pTPI+3-GFP | | TPI fwd | | ctgaggatccgatggctcgcactttcttcgtcggtg | BamHI | potef-GFP-Ala_6_-MMXN |
|  |  | TPI+3-GFP rev | | ctgaccatggctagtcaagcgttagcgttgacgata | NcoI |  |
| pTPI+9-GFP | | TPI fwd (MG579) | | ctgaggatccgatggctcgcactttcttcgtcggtg | BamHI | potef-GFP-Ala_6_-MMXN |
|  |  | TPI+9-GFP rev | | ctgaccatggcgccagctagtcaagcgttagcgttgacga | NcoI |  |
| pTPI+24-GFP | | TPI fwd | | ctgaggatccgatggctcgcactttcttcgtcggtg | BamHI | potef-GFP-Ala_6_-MMXN |
|  |  | TPI+24-GFP rev | | ctgaccatggcgatcctagccgactggccagctagt | NcoI |  |
| pmCherry-TAACTA-GFP | | TAACTA fwd | | ctgaacgcgtgtaactaatggtgagcaagggcgaggagctgt | MluI | potef-mCherry-Ala_6_-MMXN |
|  |  | GFP rev | | gatctctagactacttgtacagctcgtccatgccgaga | Xbal |  |
| pmCherry-TAGCTA-GFP | | TAGCTA fwd | | ctgaacgcgtgtagctaatggtgagcaagggcgaggagctgt | MluI | potef-mCherry-Ala_6_-MMXN |
|  |  | GFP rev | | gatctctagactacttgtacagctcgtccatgccgaga | Xbal |  |
| pmCherry-TGACTA-GFP | | TGACTA fwd | | ctgaacgcgtgtgactaatggtgagcaagggcgaggagctgt | MluI | potef-mCherry-Ala_6_-MMXN |
|  |  | GFP rev | | gatctctagactacttgtacagctcgtccatgccgaga | Xbal |  |
| pmCherry-TGACTC-GFP | | TGACTC fwd | | ctgaacgcgtgtgactcatggtgagcaagggcgaggagctgt | MluI | potef-mCherry-Ala_6_-MMXN |
|  |  | GFP rev | | gatctctagactacttgtacagctcgtccatgccgaga | Xbal |  |
| pmCherry-TGACTG-GFP | | TGACTG fwd | | ctgaacgcgtgtgactgatggtgagcaagggcgaggagctgt | MluI | potef-mCherry-Ala_6_-MMXN |
|  |  | GFP rev | | gatctctagactacttgtacagctcgtccatgccgaga | Xbal |  |
| pmCherry-TGACTT-GFP | | TGACTT fwd | | ctgaacgcgtgtgacttatggtgagcaagggcgaggagctgt | MluI | potef-mCherry-Ala_6_-MMXN |
|  |  | GFP rev | | gatctctagactacttgtacagctcgtccatgccgaga | Xbal |  |
| pmCherry-TGACAA-GFP | | TGACAA fwd | | ctgaacgcgtgtgacaaatggtgagcaagggcgaggagctgt | MluI | potef-mCherry-Ala_6_-MMXN |
|  |  | GFP rev | | gatctctagactacttgtacagctcgtccatgccgaga | Xbal |  |
| pmCherry-TGACCA-GFP | | TGACCA fwd | | ctgaacgcgtgtgaccaatggtgagcaagggcgaggagctgt | MluI | potef-mCherry-Ala_6_-MMXN |
|  |  | GFP rev | | gatctctagactacttgtacagctcgtccatgccgaga | Xbal |  |
| pmCherry-TGACGA-GFP | | TGACGA fwd | | ctgaacgcgtgtgacgaatggtgagcaagggcgaggagctgt | MluI | potef-mCherry-Ala_6_-MMXN |
|  |  | GFP rev | | gatctctagactacttgtacagctcgtccatgccgaga | Xbal |  |
| pmCherry-TGAATA-GFP | | TGAATA fwd | | ctgaacgcgtgtgaataatggtgagcaagggcgaggagctgt | MluI | potef-mCherry-Ala_6_-MMXN |
|  |  | GFP rev | | gatctctagactacttgtacagctcgtccatgccgaga | Xbal |  |
| pmCherry-TGAGTA-GFP | | TGAGTA fwd | | ctgaacgcgtgtgagtaatggtgagcaagggcgaggagctgt | MluI | potef-mCherry-Ala_6_-MMXN |
|  |  | GFP rev | | gatctctagactacttgtacagctcgtccatgccgaga | Xbal |  |
| pmCherry-TGATTA-GFP | | TGATTA fwd | | ctgaacgcgtgtgattaatggtgagcaagggcgaggagctgt | MluI | potef-mCherry-Ala_6_-MMXN |
|  |  | GFP rev | | gatctctagactacttgtacagctcgtccatgccgaga | Xbal |  |
| pGFP-PTS1 (Art1) | | PTS1 (Art1) 5’ | | cgcgtgaacggcgtcaaagcaatcctcgacagtgtgtatcagtcactagcgcacgagcggctgtaat | MluI, XbaI | potef-GFP-Ala_6_-MMXN |
|  |  | PTS1 (Art1) 3’ | | ctagattacagccgctcgtgcgctagtgactgatacacactgtcgaggattgctttgacgccgttca |  |  |
| pGFP-PTS1 (Rpe1) | | PTS1 (Rpe1) 5’ | | cgcgtgagtatcacgcctgctgccagtgcggtcgagggtaaatcacttgcgagacggccggctaagctttgat | MluI, XbaI | potef-GFP-Ala_6_-MMXN |
|  |  | PTS1 (Rpe1) 3’ | | ctagatcaaagcttagccggccgtctcgcaagtgatttaccctcgaccgcactggcagcaggcgtgatactca |  |  |
| pGFP-PTS1 (Idp1) | | PTS1 (Idp1) 5’ | | cgcgtaaagctccagactcgcggcatcgaggctggcaagctttgat | MluI, XbaI | potef-GFP-Ala_6_-MMXN |
|  |  | PTS1 (Idp1) 3’ | | ctagatcaaagcttgccagcctcgatgccgcgagtctggagcttta |  |  |
| pHA-LDH+Myc | | HA-LDH fwd | | atatggatccatgtacccatacgatgttccagattacgcagcaactcttaaggaaaaactc | BamHI | pcDNA^TM^3.1 |
|  |  | LDH+Myc rev | | atatgaattcttacaggtcctcctcgctaatgagtttctgctctcacaggtcttttaggtccttc | EcoRI |  |
| pHA-LDH-Myc | | HA-LDH fwd | | atatggatccatgtacccatacgatgttccagattacgcagcaactcttaaggaaaaactc | BamHI | pcDNA^TM^3.1 |
|  |  | LDH-Myc rev | | atatgaattcttacaggtcctcctcgctaatgagtttctgctccaggtcttttaggtccttctgg | EcoRI |  |
| pHA-LDH-TGACTA-Myc | | HA-LDH fwd | | atatggatccatgtacccatacgatgttccagattacgcagcaactcttaaggaaaaactc | BamHI | pcDNA^TM^3.1 |
|  |  | LDH+TGACTA-Myc rev | | atatgaattcttacaggtcctcctcgctaatgagtttctgctctagtcacaggtcttttaggtcc | EcoRI |  |
| pHA-LDH-TGA-18-Myc | | HA-LDH fwd | | atatggatccatgtacccatacgatgttccagattacgcagcaactcttaaggaaaaactc | BamHI | pcDNA^TM^3.1 |
|  |  | LDH-TGA-18-Myc rev | | atatgaattcttacaggtcctcctcgctaatgagtttctgctccagcctagagctcactagtca | EcoRI |  |
| pHA-LDH-TAACTA-Myc | | HA-LDH fwd | | atatggatccatgtacccatacgatgttccagattacgcagcaactcttaaggaaaaactc | BamHI | pcDNA^TM^3.1 |
|  |  | LDH-TAACTA-Myc rev | | atatgaattcttacaggtcctcctcgctaatgagtttctgctctagttacaggtcttttaggtcc |  |  |
| pHA-LDH-TAGCTA-Myc | | HA-LDH fwd | | atatggatccatgtacccatacgatgttccagattacgcagcaactcttaaggaaaaactc | BamHI | pcDNA^TM^3.1 |
|  |  | LDH-TAACTA-Myc rev | | atatgaattcttacaggtcctcctcgctaatgagtttctgctctagctacaggtcttttaggtcc |  |  |
| pGFP-TGACTA-Myc | | TGACTA-Myc 5‘ | | aattcaaaggacctaaaagacctgtgactagagcagaaactcattagcgaggaggacctgtaag | BamHI, EcoRI | pEGFP-C1 |
|  |  | TGACTA-Myc 3‘ | | gatccttacaggtcctcctcgctaatgagtttctgctctagtcacaggtcttttaggtcctttg |  |  |
| pGFP-TGACTT-Myc | | TGACTT-Myc 5‘ | | aattcaaaggacctaaaagacctgtgacttgagcagaaactcattagcgaggaggacctgtaag | BamHI, EcoRI | pEGFP-C1 |
|  |  | TGACTT-Myc 3‘ | | gatccttacaggtcctcctcgctaatgagtttctgctcaagtcacaggtcttttaggtcctttg |  |  |
| pGFP-TGACTC-Myc | | TGACTC-Myc 5‘ | | aattcaaaggacctaaaagacctgtgactcgagcagaaactcattagcgaggaggacctgtaag | BamHI, EcoRI | pEGFP-C1 |
|  |  | TGACTC-Myc 3‘ | | gatccttacaggtcctcctcgctaatgagtttctgctcgagtcacaggtcttttaggtcctttg |  |  |
| pGFP-TGACTG-Myc | | TGACTG-Myc 5‘ | | aattcaaaggacctaaaagacctgtgactggagcagaaactcattagcgaggaggacctgtaag | BamHI, EcoRI | pEGFP-C1 |
|  |  | TGACTG-Myc 3‘ | | gatccttacaggtcctcctcgctaatgagtttctgctccagtcacaggtcttttaggtcctttg |  |  |
| pGFP-TGACCA-Myc | | TGACCA-Myc 5‘ | | aattcaaaggacctaaaagacctgtgaccagagcagaaactcattagcgaggaggacctgtaag | BamHI, EcoRI | pEGFP-C1 |
|  |  | TGACCA-Myc 3‘ | | gatccttacaggtcctcctcgctaatgagtttctgctctggtcacaggtcttttaggtcctttg |  |  |
| pGFP-TGACAA-Myc | | TGACAA-Myc 5‘ | | aattcaaaggacctaaaagacctgtgacaagagcagaaactcattagcgaggaggacctgtaag | BamHI, EcoRI | pEGFP-C1 |
|  |  | TGACAA-Myc 3‘ | | gatccttacaggtcctcctcgctaatgagtttctgctcttgtcacaggtcttttaggtcctttg |  |  |
| pGFP-TGACGA-Myc | | TGACGA-Myc 5‘ | | aattcaaaggacctaaaagacctgtgacgagagcagaaactcattagcgaggaggacctgtaag | BamHI, EcoRI | pEGFP-C1 |
|  |  | TGACGA-Myc 3‘ | | gatccttacaggtcctcctcgctaatgagtttctgctctcgtcacaggtcttttaggtcctttg |  |  |
| pGFP-TGAATA-Myc | TGAATA-Myc 5‘ | | aattcaaaggacctaaaagacctgtgaatagagcagaaactcattagcgaggaggacctgtaag | | BamHI, EcoRI | pEGFP-C1 |
|  | TGA ATA-Myc 3‘ | | gatccttacaggtcctcctcgctaatgagtttctgctctattcacaggtcttttaggtcctttg | |  |  |
| pGFP-TGATTA-Myc | TGATTA-Myc 5‘ | | aattcaaaggacctaaaagacctgtgattagagcagaaactcattagcgaggaggacctgtaag | | BamHI, EcoRI | pEGFP-C1 |
|  | TGATTA-Myc 3‘ | | gatccttacaggtcctcctcgctaatgagtttctgctctaatcacaggtcttttaggtcctttg | |  |  |
| pGFP-TGAGTA-Myc | TGAGTA-Myc 5‘ | | aattcaaaggacctaaaagacctgtgagtagagcagaaactcattagcgaggaggacctgtaag | | BamHI, EcoRI | pEGFP-C1 |
|  | TGAGTA-Myc 3‘ | | gatccttacaggtcctcctcgctaatgagtttctgctctactcacaggtcttttaggtcctttg | |  |  |
| pmCherry-SKL | mCherry-SKL 5‘ | | gatccttacagcttggatagag | | BamHI, EcoRI | pmCherry-C1 |
|  | mCherry-SKL 3‘ | | aattctctatccaagctgtaag | |  |  |
| pEGFP-LDH | GFP-LDH fwd | | atatgaattccatggcaactcttaaggaaaaactc | | EcoRI | pEGFP-C1 |
|  | LDH rev | | atatggatcctcacaggtcttttaggtccttc | | BamHI |  |
| pEGFP-LdhB_Pex_ | GFP-LDH fwd | | atatgaattccatggcaactcttaaggaaaaactc | | EcoRI | pEGFP-C1 |
|  | LDH+PTS1 rev | | atatggatccaggctttgattctgtgagccc | | BamHI |  |
| pEGFP-LdhB_Cyt_ | GFP-LDH fwd | | atatgaattccatggcaactcttaaggaaaaactc | | EcoRI | pEGFP-C1 |
|  | LDH- PTS1 rev | | atatggatccctacagcctagagctcactagccacaggtcttttagg | | BamHI |  |
| pEGFP-Mdh1 | GFP-MDH fwd | | atatgaattccatgtctgaaccaatcagagtcc | | EcoRI | pEGFP-C1 |
|  | MDH rev | | atatggatcctcaggcagaggaaagaaattcaaaagc | | BamHI |  |
| pEGFP-Mdh1_Pex_ | GFP-MDH fwd | | atatgaattccatgtctgaaccaatcagagtcc | | EcoRI | pEGFP-C1 |
|  | MDH+PTS1 rev | | atatggatccactgtcattcacaaacctgtacc | | BamHI |  |
| pEGFP-Mdh1_Cyt_ | GFP-MDH fwd | | atatgaattccatgtctgaaccaatcagagtcc | | EcoRI | pEGFP-C1 |
|  | MDH-PTS1 rev | | atatggatcctcaaagacgacatttagattcttcagctttgaagcatttagtaacatcattgtctagccaggcagaggaaagaaattc | | BamHI |  |
| pEGFP-PTS1 (Mdh1) | PTS1-MDH-Sense | | aattctaaatgcttcaaagctgaagaatctaaatgtcgtctttgag | | EcoRI | pEGFP-C1 |
|  | PTS1-MDH-Antisense | | gatcctcaaagacgacatttagattcttcagctttgaagcatttag | | BamHI |  |
| pEGFP-PTS1 (LdhB) | PTS1-LDH-Sense | | aattctgacctaaaagacctgtggctagtgagctctaggctgtagg | | EcoRI | pEGFP-C1 |
|  | PTS1-LDH-Antisense | | gatccctacagcctagagctcactagccacaggtcttttaggtcag | | BamHI |  |
| pEGFP-PTS1 (Ppa2) | PTS1-PPA2-Sense | | aattctaaacatctgaaattctgctgtcaagattcccatctctaag | | EcoRI | pEGFP-C1 |
|  | PTS1-PPA2-Antisense | | gatccttagagatgggaatcttgacagcagaatttcagatgtttag | | BamHI |  |
